# Model-Informed Risk Assessment and Decision Making for an Emerging Infectious Disease in the Asia-Pacific Region

**DOI:** 10.1371/journal.pntd.0005018

**Published:** 2016-09-23

**Authors:** Robert Moss, Roslyn I. Hickson, Jodie McVernon, James M. McCaw, Krishna Hort, Jim Black, John R. Madden, Nhi H. Tran, Emma S. McBryde, Nicholas Geard

**Affiliations:** 1 Centre for Epidemiology and Biostatistics, Melbourne School of Population Health, The University of Melbourne, Melbourne, Australia; 2 IBM Research - Australia, Melbourne, Australia; 3 Murdoch Childrens Research Institute, Royal Children’s Hospital, Melbourne, Australia; 4 School of Mathematics and Statistics, The University of Melbourne, Melbourne, Australia; 5 Nossal Institute, Melbourne School of Population and Global Health, The University of Melbourne, Melbourne, Australia; 6 Centre of Policy Studies, Victoria University, Melbourne, Australia; 7 Department of Medicine, The University of Melbourne, Melbourne, Australia; 8 Australian Institute of Tropical Health and Medicine, James Cook University, Townsville, Australia; University of Florida, UNITED STATES

## Abstract

**Background:**

Effective response to emerging infectious disease (EID) threats relies on health care systems that can detect and contain localised outbreaks before they reach a national or international scale. The Asia-Pacific region contains low and middle income countries in which the risk of EID outbreaks is elevated and whose health care systems may require international support to effectively detect and respond to such events. The absence of comprehensive data on populations, health care systems and disease characteristics in this region makes risk assessment and decisions about the provision of such support challenging.

**Methodology/principal findings:**

We describe a mathematical modelling framework that can inform this process by integrating available data sources, systematically explore the effects of uncertainty, and provide estimates of outbreak risk under a range of intervention scenarios. We illustrate the use of this framework in the context of a potential importation of Ebola Virus Disease into the Asia-Pacific region. Results suggest that, across a wide range of plausible scenarios, preemptive interventions supporting the timely detection of early cases provide substantially greater reductions in the probability of large outbreaks than interventions that support health care system capacity after an outbreak has commenced.

**Conclusions/significance:**

Our study demonstrates how, in the presence of substantial uncertainty about health care system infrastructure and other relevant aspects of disease control, mathematical models can be used to assess the constraints that limited resources place upon the ability of local health care systems to detect and respond to EID outbreaks in a timely and effective fashion. Our framework can help evaluate the relative impact of these constraints to identify resourcing priorities for health care system support, in order to inform principled and quantifiable decision making.

## Author Summary

Low and middle income countries face a serious challenge when confronting emerging infectious disease (EID) threats. Their risk of experiencing outbreaks can be greater than in many high income countries, while their capacity to respond effectively may be constrained by competing demands on limited health care system resources. The globalised nature of health security argues for international support to improve local health care systems, but limited data makes risk assessment and decision making difficult. We propose a mathematical modelling framework that can help explore a variety of outbreak and intervention scenarios. Our framework can assist with the identification of constraints that limit the ability of local health care systems to detect and respond to EID outbreaks in a timely and effective fashion, and assess the relative importance of these constraints to help establish priorities for health care system support. We illustrate the use of our framework by considering the importation of Ebola into the Asia-Pacific region, with results emphasising the critical role played by effective surveillance in controlling localised outbreaks.

## Introduction

The Ebola Virus Disease (EVD) outbreak in West Africa highlighted the ongoing threat to global health posed by emerging infectious diseases (EIDs). As the outbreak waned, attention has turned towards the lessons to be learned for future EID preparedness and response [1]. In particular, the prolonged delay in international response reinforced the importance of ensuring that local health care systems are capable of detecting and responding to localised outbreaks before they reach epidemic proportions [2,3].

The prospect of EID outbreaks in low and middle income countries raises particular concerns and challenges for global health agencies. The risk of emergence of novel pathogens in many of these countries is elevated [4], and there may be limited capacity to mount an effective response [5]. Greater global connectivity increases both the risks and international consequences of EID outbreaks in these countries [6]. In consequence, there is a growing recognition that health security is a shared responsibility and that supporting health care systems capacity in low and middle income countries is a necessary step towards improving global health security.

A significant challenge facing international health agencies responding to EID outbreaks in the Asia-Pacific region is the scarcity of data required for risk assessment and decision making [5,7]. These challenges are compounded when attempting to formulate plans prior to an outbreak occurring, when epidemiological data relevant to a specific country may not exist or be readily available to researchers, policy makers and response personnel. Despite these challenges, analysing potential outbreak risks is essential for timely decision making about effective preparedness and response strategies.

Here we demonstrate how mathematical modelling can support risk assessment and decision making about preparedness for and response to an EID outbreak [8], focusing on the role of health care system support and behavioural interventions in outbreak control. We illustrate this application of mathematical modelling using the potential importation of an active EVD case into the Asia-Pacific region as a case study. Although no such importation has occurred to date, the risk has been recognised [9], prompting consideration of the likelihood of an outbreak occurring and evaluating alternative preparedness and response strategies. Furthermore, other viral haemorrhagic fevers—both external to the Asia-Pacific region, such as Marburg virus disease [10] and Lassa fever [11], and those already present, such as Nipah virus [12]–may have similar pandemic potential to EVD, and the model framework introduced here would be directly applicable.

We show how to use this framework, in the face of uncertainty, to identify key drivers of outbreak controllability and the most effective resourcing strategies to enhance preparedness and response. Our results suggest that early detection and ongoing ascertainment of cases have the most substantial impact on controllability across a wide range of demographic, health care system and intervention scenarios. However, we show how priorities for health care system support depend upon the local context. We compare our findings to results from qualitative assessments of EVD risk undertaken by WHO [13,14], and conclude by discussing the implications for EVD preparedness in particular, and the broader utility of mathematical modelling for EID risk assessment and decision making in data-poor scenarios.

### The 2014–2015 West African Ebola virus disease outbreak

EVD was first identified in Zaire in 1976 and there have since been at least twenty-five outbreaks in Africa. The 2014 West African outbreak—first reported in Guinea and subsequently becoming established in Liberia and Sierra Leone—is the largest to date. Symptoms of EVD include vomiting, diarrhoea and haemorrhaging, and transmission occurs via direct contact with blood and other bodily fluids. It is characterised by an incubation period of up to three weeks during which onward transmission is unlikely, followed by increasingly severe symptoms and associated infectiousness. Case fatality rates are high, and improperly buried bodies represent an ongoing source of infection [15]. In the absence of widely available pharmaceutical measures for treatment or prevention during the 2014 West African outbreak, control efforts centred around identification and isolation of cases, tracing of known contacts, and hygienic burial of people who died from EVD.

Unlike previous outbreaks, which were largely confined to rural areas, the 2014 West African outbreak spread to urban regions, which contributed to the greater number of cases: official estimates are approximately 28,000 cases with over 11,000 deaths, although such numbers may have underestimated the true magnitude [16]. Health care workers were disproportionately represented among cases and the full costs of the outbreak in terms of the impact on health infrastructure are still being realised [2]. During the outbreak, there was considerable international concern about the potential for the global transmission of EVD, heightened by the appearance of cases in Spain, the United Kingdom and the United States of America [17].

### The Asia-Pacific region

Modern patterns of global transport mean that no country is isolated from the risk of disease importation, as demonstrated by the rapid worldwide spread of H1N1 influenza in 2009 [18]. The Asia-Pacific region is of particular concern, with Southeast Asia termed a ‘hotspot’ for EIDs due to social and environmental factors supporting the evolution and transmission of novel pathogens [5].

**Fig 1:**
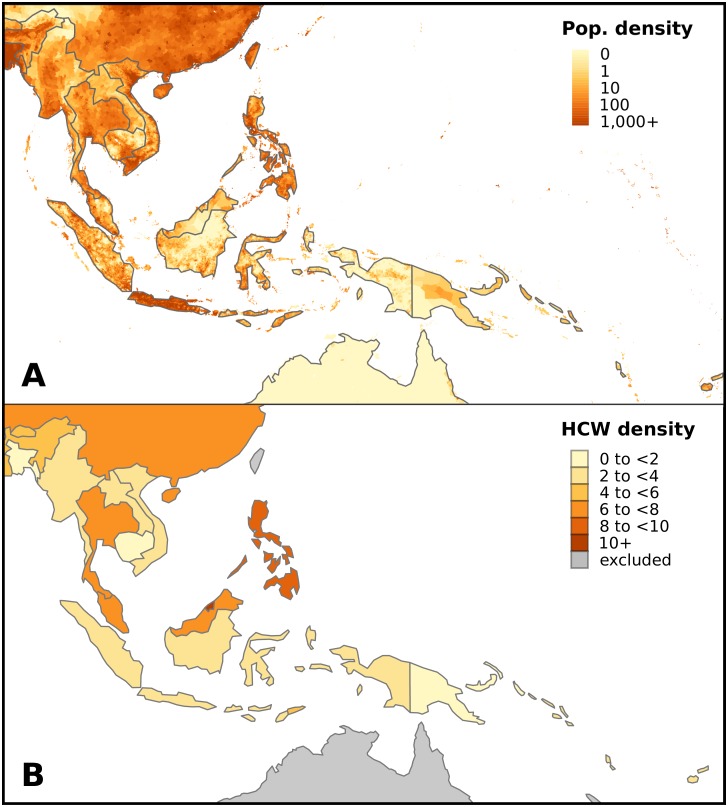
Population and health care system diversity in Southeast Asia and the Western Pacific. **(A)** Population density ranges from less than 1 person per square kilometre in parts of Papua New Guinea and Indonesia to over 6,800 people per square kilometre in Singapore. Data obtained from Center for International Earth Science Information Network (CIESIN), Columbia University (http://sedac.ciesin.columbia.edu/gpw). **(B)** Number of health care workers per thousand population ranges from 1.2 in Cambodia to 11.5 in Brunei Darussalam. Data obtained from Global Health Observatory data repository (http://apps.who.int/gho/data/).

The considerable diversity—geographic, demographic, cultural and economic—of Asia-Pacific countries means that risks associated with EIDs will vary greatly across the region, both between and within countries (Fig 1). Differences in health care system funding, access and infrastructure will influence capacity to detect and respond to EID outbreaks, as evidenced by recent experience of avian influenza H5N1, pandemic influenza H1N1 and severe acute respiratory syndrome (SARS).

Many countries in this region are understudied with respect to their health infrastructure and there is a poor understanding of their response capacity in the event of an EID outbreak. World Health Organization (WHO) regional offices in the Western Pacific (WPRO) and South East Asia (SEARO) have assessed the need to build national capacities to undertake surveillance, infection prevention and control, and public health emergency preparedness [19]. Annual assessments of these capacities are self-reported, and provide only a general statement of accomplishment [20].

The risks associated with EVD spreading to Southeast Asia were recognised during the 2014 West African outbreak [9]. In addition to global air passenger flows, specific vectors of risk included the presence of miners and aid workers in West Africa with family ties to various countries in Southeast Asia. In September 2014, WHO regional offices undertook a rapid assessment of EVD virus preparedness across countries in the region, followed by a simulation exercise to train public health staff. A risk assessment for EVD infection in the region was undertaken using the WHO guidance for rapid risk assessment of acute public health events [14]. In addition, and of particular relevance to potential risks, forty health professionals from India and Bangladesh participated in teams supporting outbreak control in West Africa, as well as staff from Pacific Island Countries through the West Pacific Ebola Support Team [20].

Despite the ongoing efforts by WHO, the EVD preparedness review identified that all thirteen surveyed Asian countries, but only nine of thirteen surveyed Pacific Island countries, had health facilities designated to isolate suspected or confirmed cases. Only two of the Pacific Island countries surveyed reported adequate supplies of personal protective equipment (PPE) for EVD response and containment. In addition, EVD surveillance protocols had been developed and disseminated in all but one of thirteen Asian countries, but only two of thirteen Pacific countries [13,21].

In the presence of competing demands for attention and resources, there is a risk that efforts to improve health policies and systems will be reactive and transient in nature [9]. This reinforces the importance of ongoing support from international and regional organisations, such as the Association of Southeast Asian Nations (ASEAN) and WHO, but identifying the most effective forms of such support across this diverse region is a formidable challenge.

### Modelling of infectious diseases

Mathematical modelling can be a powerful tool to inform decision making in data-poor scenarios [22,23]. Models can incorporate data that is available from a range of quantitative and qualitative epidemiological and sociological sources. Even when precise predictions are not possible, models can provide ‘best estimates’ that enable decision makers to rapidly evaluate alternative outbreak, surveillance and response scenarios. The process of constructing a mathematical model requires assumptions about drivers of disease transmission to be made explicit, aiding transparency. Uncertainties in current knowledge can be incorporated into parameter estimates, and can help to provide estimates of the risk of various possible outcomes [24–26]. Finally, mathematical models can enable identification and prioritisation of key data requirements, providing arguments and evidence to guide future data collection efforts.

Mathematical models were used extensively during the 2014 West African EVD outbreak [27], including in the early stages when there was considerable uncertainty around disease activity. A key aim was to project the possible course of the outbreak and assess the potential effectiveness of alternative interventions within West Africa [28–33]. Models were also used to estimate the risk of global transmission [34,35].

## Methods

We used mathematical modelling to explore hypothetical scenarios that might result if an active EVD case were to be imported to the Asia-Pacific. The two key questions motivating the construction of our model were:

1. If an active EVD case travelled from an infected country to the Asia-Pacific region, what is the risk of an outbreak subsequently occurring?
2. What are the key health care system constraints that limit the ability of a local health care system to detect and respond to an EVD outbreak in a timely fashion and what type of international intervention would most effectively address these constraints?

To construct a model capable of addressing these questions, the following data requirements were identified:

1. characteristics of EVD relevant to infection transmission (disease characteristics);
2. demographic, sociological and behavioural characteristics of the target populations relevant to the transmission of EVD, and their variation across the region (population characteristics);
3. the current capacity of local health care systems to detect and respond to importation and transmission of EVD, and their variation across the region (health care system characteristics); and
4. plausible interventions to support local health care systems to detect and respond to importation and transmission of EVD (interventions).

These data requirements are addressed in the following subsections. We then describe appropriate outcome measures given the data-poor situation of interest, and outline the specific scenarios explored in this paper.

### Disease characteristics

The natural history of EVD has been well characterised from previous outbreaks, and disease characteristics such as the basic reproduction number and the duration of the latent and infectious periods were therefore available from previous epidemiological studies [15].

**Fig 2:**
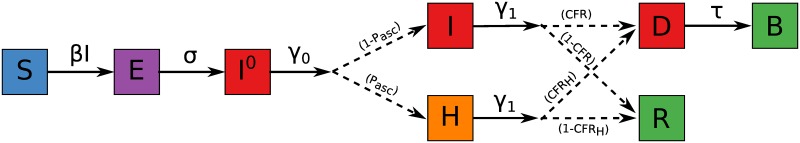
The Ebola model. The natural history of infection comprises susceptibility (S), exposure (E) at a rate determined by the force of infection β and the current prevalence of infectious individuals and unburied dead bodies. Exposed individuals progress to mild infectiousness prior to developing symptoms (I0) at rate σ, and symptomatic infection (I) at rate γ_0_, followed by either death (D) or recovery (R) at rate γ_1_. The proportion of infections leading to death or recovery is informed by estimates of the case fatality ratio (CFR). Dead bodies remain infectious prior to burial (B) at rate τ. Ascertainment of cases (with probability p_asc_) allows symptomatic individuals to be hospitalised in isolation wards (H), which reduces their contribution to transmission and increases their probability of recovery. Full equations describing the model are provided in Model description in S1 Methods.

We used a stochastic SEIR-type model (Fig 2), with the addition of compartments for the number of unburied dead bodies (which form a significant source of transmission, if there are interactions with the dead body, as is common in various funeral rites) and the number of safely buried dead bodies (assumed to no longer contribute to transmission). All individuals are initially susceptible to infection (S). Once infected, they enter a latent incubation stage (E), from which they transition to become infectious, but not yet symptomatic (I_0_). At the point of developing symptoms (I), their infectiousness increases. Post-infection, individuals either recover and are no longer infectious (R) or die and remain infectious (D) until buried (B).

We also introduced a compartment (H) for individuals who have become symptomatic and are visible to the health care system. Separating these visible (i.e., ascertained) cases from the remaining, undetected cases (I) allows the model to incorporate actions such as case isolation (thereby decreasing their ability to infect others) and the provision of treatment (thereby decreasing their risk of dying). Full details of the model are provided in Model description in S1 Methods.

### Population characteristics

While many socio-cultural factors may influence the frequency and intensity of contact between individuals that facilitate the spread of infection, we used a simple distinction between rural and urban populations that proved to be an important determinant of EVD transmission [29]. We distinguish rural and urban populations in the model by allowing more transmission from infectious individuals in urban populations than in rural populations.

Another key driver of EVD transmission is the frequency and intensity of contact between healthy individuals and infectious dead bodies as a consequence of burial practices, which may vary according to the dominant religion. For example, Islamic practices include burying the dead body as soon as possible, while in non-Islamic regions the burial may be delayed for a number of days in order to give distant family members time to visit the deceased. Other cultural traditions may also facilitate substantial transmission after death, such as the Papua New Guinean ritual of ‘haus krai’, where the body is typically returned to the home village for burial, after which the extended family and friends gather for a wake that can continue for many days. Funerals and burials have been recognised as potential ‘super-spreading’ events [36], which may result in a substantially greater than expected number of secondary cases [37]. The frequency of these events may therefore influence the likelihood of an outbreak occurring.

In the model, we characterised the different burial practices by the mean duration of the pre-burial period (i.e., the average delay between death and burial) and the daily force of infection exerted by dead bodies (i.e., the degree of interaction between healthy individuals and the body). The parameter values were informed by expert knowledge of burial customs (where available), by knowledge of the dominant religions in a given region, and from estimates of the force of infection from the West African outbreak.

### Health care system characteristics

A country’s health care system includes people and institutions responsible and resources available for disease surveillance—case ascertainment and contact tracing—and isolation and treatment of detected cases.

Surveillance capacity was represented in the model by two distinct quantities: the number of transmission events that occurred prior to the *first detected case*; and the *case ascertainment probability* of subsequent symptomatic cases. Limited contact tracing was also included, allowing some contacts of identified cases to be *monitored* and therefore experience a higher probability of being identified should they subsequently become infected.

We used the additional model compartment (H) to represent the isolation of identified cases in hospital isolation wards, as depicted in Fig 2. Hospital isolation was assumed to prevent onward transmission and to increase survival probability. Hospital isolation and contact tracing were both subject to capacity constraints imposed by limited resource and workforce availability.

We also stratified the model population into health care workers (HCWs) and the general community, to allow differential risks of exposure and case ascertainment. Prior to the first detected case, HCWs were assumed to experience a greater risk of exposure than the general community, due to close contact in medical settings and a lack of recognition of the need for special precautions. Subsequent to the first detected case, HCWs were assumed to experience a lower risk of exposure than the general community, due to the adoption of appropriate infection control measures. At this stage, any cases among HCWs were assumed to be automatically detected.

This stratification also explicitly captured the impact of the availability of the health care workforce (i.e., uninfected HCWs, assumed to be capable of providing health services) on health care capacity. Depletion of this workforce (due to infection) was assumed to reduce the provision of case ascertainment, contact tracing and monitoring, and case isolation. Specifically, only 50% of health care capacities were assumed to be available when 90% of the HCWs were available, and no health care capacities were available once 80% or less of the HCWs remained available (see Fig 2 in S1 Methods). These values are in broad agreement with the observed impact on health care systems in Sierra Leone, where HCW mortality of around 20% was accompanied by substantial loss of trust in the health care system, substantial decreases in all-cause consultations and admissions and health care productivity and effectiveness was diminished amongst both employed and volunteer personnel [38].

The health care systems of Asia-Pacific countries are characterised by diversity in their level of development. Access to, and standards of, health care services can also vary substantially within a single country. While data on hospital capacity and size of the health care workforce was available, for some countries information on the geographical distribution of these resources was limited. We made two broad assumptions in calibrating our model: that health care system resources were more likely to be concentrated in urban centres, and that the remaining health care system resources were distributed within countries proportional to population density.

### Interventions

Several strategies for boosting the existing surveillance and health care capacity were identified as practical and potentially effective:

1. Enhanced syndromic disease surveillance in the general population, leading to an improved likelihood of case ascertainment.
2. Provision of additional isolation wards and health care workers; and provision of personal protective equipment (PPE) to reduce transmission within the household during home-based quarantine [29,39].

These interventions were represented by increasing the case ascertainment probability of cases in the general community, and by increasing the available capacities for contact monitoring and hospital isolation.

In addition to medical interventions, changes to social behaviours and cultural practices can reduce the force of infection in the community [15,40,41]. We identified two behavioural interventions to reduce the force of infection in the community, based on evidence of success in the West African outbreak and in other infectious disease outbreaks:

1. Reducing interaction with dead bodies, by adopting changes in funeral rites, burial practices, and the storage of dead bodies prior to burial. This was represented by decreasing the daily force of infection from unburied bodies and by decreasing the delay between death and burial.
2. Reducing infection from infectious individuals in the community by enhancing hygiene practices in the community (e.g., hand washing, use of face masks, and other practices that reduce exposure to bodily fluids). This was represented by decreasing the daily force of infection experienced by the general community.

In response to a *perceived* potential EID threat, it might be possible to deliver an intervention prior to an actual outbreak occurring, while responding to an observed outbreak would necessarily involve some delay. It is therefore critical to identify how the required *magnitude* of an intervention to reach an “acceptable” probability of outbreak control depends upon the *timing* of that intervention.

In our sensitivity analysis, we explored how both the *magnitude* and the *timing* of these interventions affected the population experience of disease. Intervention timing (“delay”) was defined as the time at which the increased capacities and/or decreased forces of infection were realised, relative to the time at which the *first identified case* was detected. Accordingly, a delay of zero weeks meant that the intervention was deployed prior to the first case being identified. The first identified case was defined relative to the cumulative number of infectious individuals that became symptomatic (i.e., transitioned to the I model compartment).

### Outcome measures

In the absence of a health care response the distribution of outbreak sizes was bimodal, consistent with classic results from stochastic infection theory [42]. Around a quarter of the model simulations produced outbreaks of 16 cases or fewer, representing stochastic fade-out of the disease. The remaining simulations yielded outbreaks with attack rates of around 80%, representing uncontrolled outbreaks. While high, it is important to note that these attack rates correspond to the hypothetical scenario in which there is no health care system or social response to the outbreak. An attack rate of 80% is consistent with theoretical estimates for an infectious disease of equivalent transmissibility to that estimated for EVD (i.e., a basic reproduction number of around 2), and with observed epidemic behaviour early in the West African outbreak [28].

**Fig 3:**
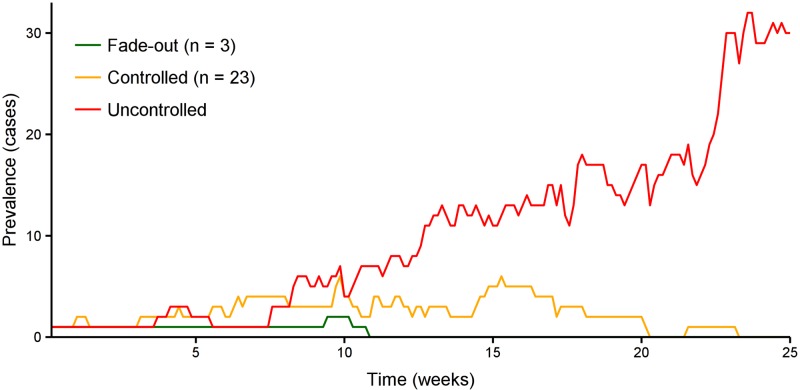
Time series of disease prevalence for three example simulations. These three examples demonstrate the three possible categories of epidemic outcome: stochastic fade-out, a controlled outbreak, and an uncontrolled outbreak.

The variety and complexity of output that a disease model provides (such as the final size distribution) can be both overwhelming and inappropriate in a decision support context. Accordingly, we established a qualitative measure to assess the outcome of an epidemic based on its final size (see Fig 3 for example outcomes):

**Fade-out (green).** For any infectious disease, when very few cases are present in a population, it is possible that these individuals will recover or die before the infection spreads further, and the outbreak will be self-limiting.
**Controlled (orange).** A controlled outbreak is one that would have been uncontrolled in the absence of local response or intervention (that is, it would not have been expected to fade out of its own accord), but whose final size was greatly diminished due to a local response or intervention.
**Uncontrolled (red).** An uncontrolled outbreak is one in which the local response measures or external interventions have limited impact on the course of the epidemic compared with the ‘no intervention’ scenario, as assessed by the final attack rate in the population.

**Fig 4:**
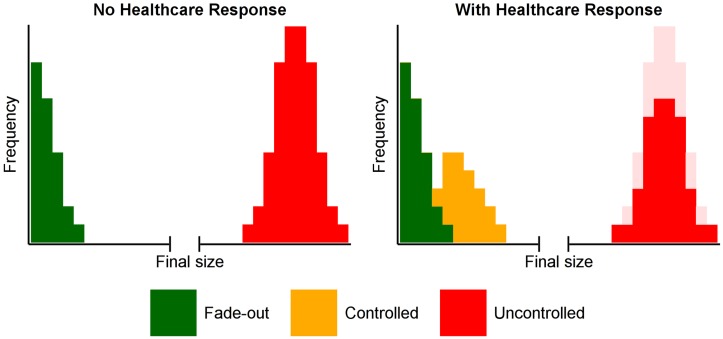
The classification of epidemic outcomes. In the absence of a health care response, EVD importation may result in zero or very few secondary cases (green, left panel) or may result in a large, uncontrolled epidemic (red, left panel). A health care response may mitigate some of these uncontrolled epidemics by greatly reducing their final size (yellow, right panel).

We classified epidemics according to the above measure, as illustrated in Fig 4. The left panel illustrates the situation in the absence of a response, where outbreaks either fade out or are uncontrolled. The right panel demonstrates how the response has little effect on outbreaks that would have faded out of their own accord, but greatly reduces the impact of a proportion of previously uncontrolled outbreaks. The distribution of outbreak sizes remained bimodal, with 96% of simulations resulting in fewer than 500 cases (fade-out and controlled epidemics) or attack rates of around 80% (uncontrolled epidemics). Accordingly, we used a threshold of 1,000 cases to distinguish between controlled and uncontrolled epidemics.

### Scenarios

Since the natural history of EVD was known to be well-characterised and stable, we were able to identify appropriate parameter values for the disease characteristics (15). For the remaining parameters, related to the proposed intervention strategies, we derived broad estimates and performed sensitivity analyses. Because disease transmission is highly stochastic in nature when there are a small number of cases, it was critical to use a stochastic transmission model. This meant that there was no single representative outbreak for a single scenario. Accordingly, for each scenario we performed 1,000 simulations to estimate the probability of each outcome.

In each of the results figures, we use a stacked bar for each scenario to represent the percentage of these simulations that were categorised as resulting in each of the three outcomes defined above (fade-out, controlled or uncontrolled). The baseline model behaviour, in the absence of any health care system response, is that all outbreaks either fade-out or are uncontrolled.

A particular country or region of interest was represented by a subset of the parameter values that best represented its (assumed) capacities and behavioural practices. To understand how this local context affected the probability of controlling an EVD outbreak, we performed a sensitivity analysis to identify how interventions might affect the probability of controlling an EVD outbreak in that country or region. For all scenarios reported below, we also explored the effects of the first identified case being the 5^th^, 10^th^, 25^th^ or 50^th^ symptomatic individual. Full parameter values for all scenarios are provided in the Simulation scenarios in S1 Methods.

## Results

As outlined in the Scenarios Section, the results represent an exploration of how variation in a region’s local response capacity, and additional interventions (either health care system support or behavioural modification) affect the controllability of an outbreak. We then use Papua New Guinea as a case study of these scenarios in the context of a specific health care system.

### Variation in local response capacity

To estimate the impact of endogenous response capacity on control, we varied the case ascertainment probability and size of the health care system, comprising the size of the health care workforce and level of available isolation and contact tracing facilities.

**Fig 5:**
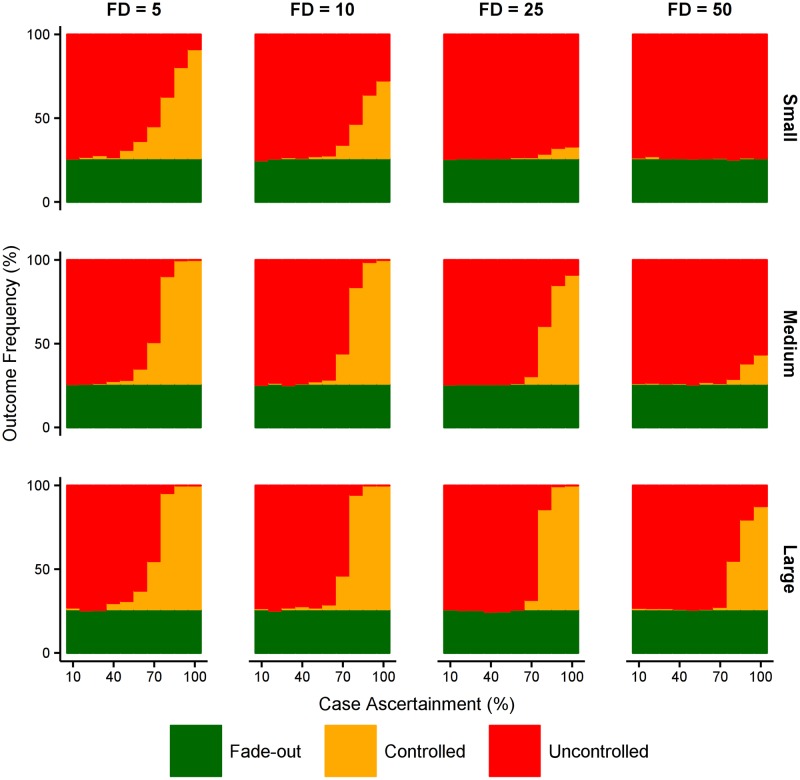
Effect of local response capacity. Epidemic outcomes as a function of case ascertainment (x-axis), subject to the first detected case (FD) and the health care system capacity (small: 0.1% of population are HCWs; medium: 0.2% of population are HCWs; large: 0.3% of population are HCWs; 1:15 ratio of HCWs to bed capacity, 1:50 ratio of HCWs to contact-tracing capacity.

The most salient conclusion is the simultaneous importance of early detection *and* high subsequent ascertainment (Fig 5). A larger health care capacity can mitigate the failure of early detection, but only to a limited degree. Controlled outbreaks only occur more frequently than uncontrolled outbreaks when case ascertainment is 80% or higher, *regardless* of health care capacity.

### Intervening to support a health care system response

To estimate the merits of providing additional support for a health response, we considered two types of interventions: increasing case ascertainment (i.e., surveillance) and increasing health care capacities.

**Fig 6:**
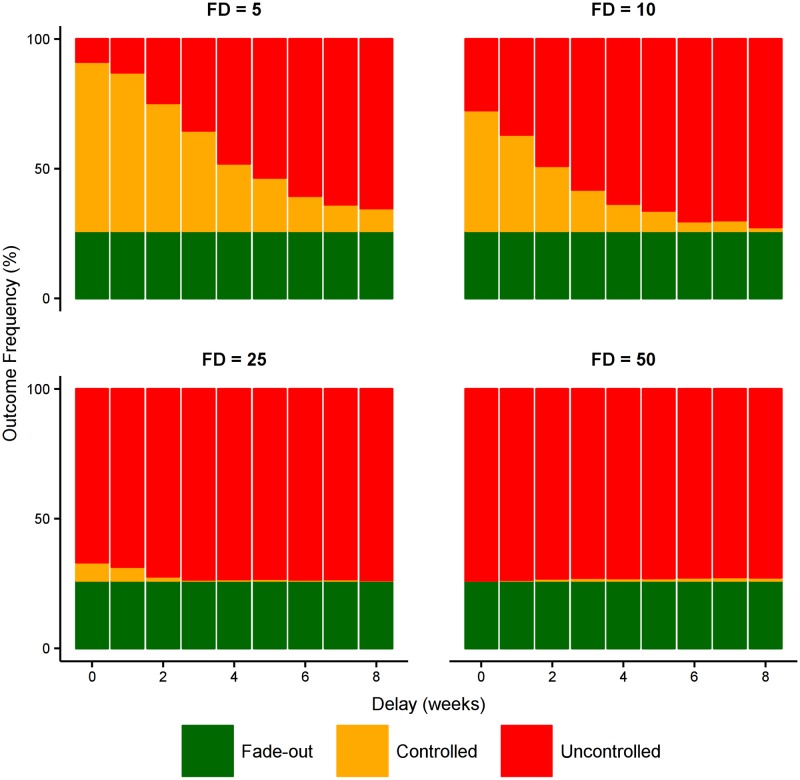
Effect of increased case ascertainment. Starting with a baseline case ascertainment of 20%, this figure shows the effect of boosting ascertainment to 100% (i.e., perfect detection) at different times after the first detected case. This clearly shows the simultaneous importance of early detection and high ascertainment; provision of one is not a substitute for lack of the other.

Reactively increasing case ascertainment *after* the first detected case, from a baseline of 20% to 100% (Fig 6) demonstrates the *simultaneous* importance of early detection and high ascertainment; both qualities are critical for outbreak control and the provision of one is not a substitute for a lack of the other. When the first case is detected early (e.g., the fifth actual case, “FD = 5”), the probability of outbreak control is 88% when case ascertainment is perfect for the duration of the outbreak. If, however, perfect case ascertainment is only achieved four weeks after the first detected case, the probability of outbreak control falls to 35%.

**Fig 7:**
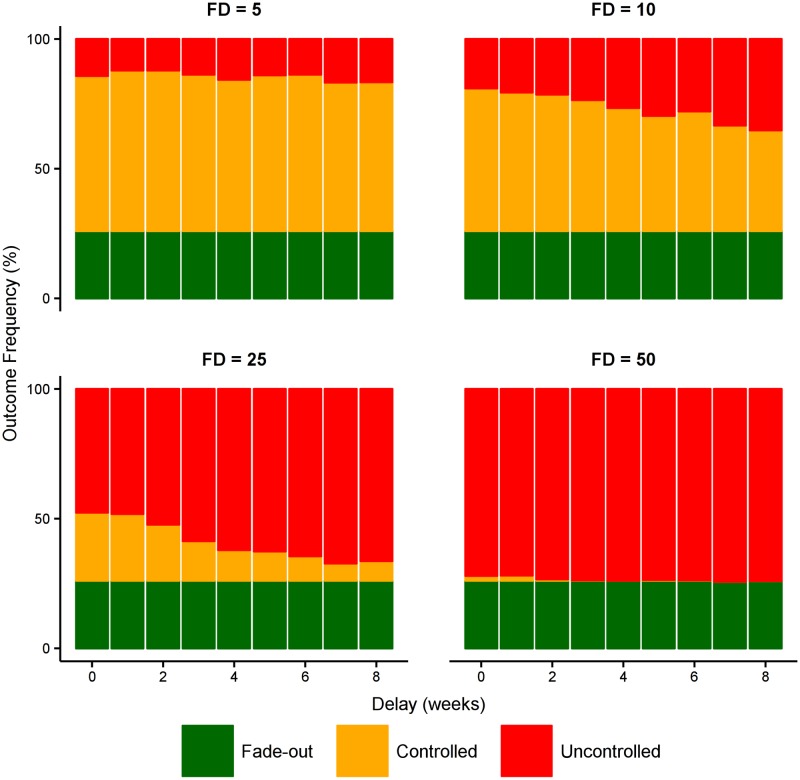
Effect of improved health care system capacity. Starting with a baseline case ascertainment of 80% and a small health care system (0.1% of population are HCWs, 1:15 and 1:50 ratio of bed and contact-tracing capacities to HCWs, as above), this figure shows the effect of doubling both the health care capacity and the health care workforce at different times after the first detected case. This increase in capacity represents the transition from a small health care system to a medium health care system, as defined in Fig 5. This clearly shows the importance of early detection; the time delay in delivering the additional capacity is less important over this timescale of 0–8 weeks, because the existing health care system is capable of accommodating patients in the early stage of the outbreak.

In contrast, where health care resources are limited, a boost in health care capacity may instead be required to increase the probability of outbreak control. As shown in Fig 7, the importance of early detection remains paramount (i.e., to trigger a rapid health care response), but the time at which the health care capacity is boosted is less important. This is in stark contrast to boosting case ascertainment, and reflects the ability of the *existing* health care system to respond effectively until the outbreak reaches a critical size. As long as the additional capacity is delivered *before* this critical size is reached, the impact of the intervention will not be diminished

### Intervening to modify behaviour

**Fig 8:**
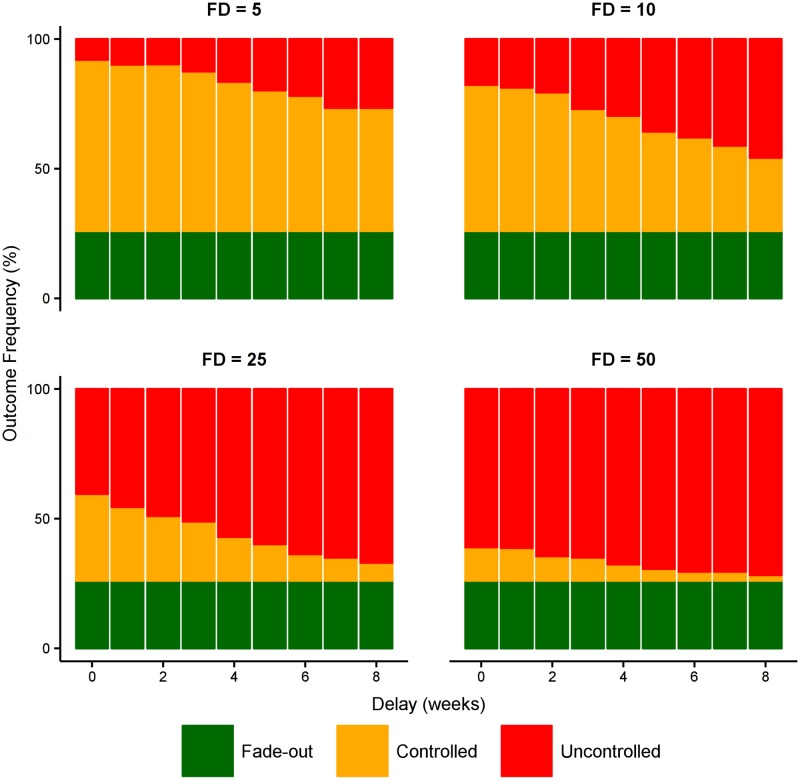
Effect of reducing force of infection from dead bodies. Starting with a baseline case ascertainment of 70% and a small health care system (0.1% of population are HCWs, 1:15 and 1:50 ratio of bed and contact-tracing capacities to HCWs, as above), this figure shows the effect of reducing the force of infection from dead bodies (β_D_) by 25%. This represents a sociocultural intervention that changes burial practices, as conducted in previous Ebola outbreaks and the temporary storage of dead bodies in the 2009 cholera outbreak in Papua New Guinea. Reducing the force of infection (whether directly or indirectly) will decrease the epidemic burden, but the likelihood of controlling the epidemic is strongly affected by the speed with which such an intervention can be delivered. Success requires both early detection and a rapid response from the community.

Reducing the force of infection from dead bodies substantially increases the likelihood of control in all scenarios (Fig 8). However, earlier detection of the first case provides greater time in which to prepare and deliver an effective *reactive* intervention. The later the first detection occurs, the more critical it becomes to deliver the intervention rapidly. When the first detection is the fifth actual case (“FD = 5”) a delay of 4 weeks before burial practices are changed reduces the likelihood of control from 89% to 78%, but when the first detection is the twenty-fifth actual case (“FD = 25”) a delay of 4 weeks before burial practices are changed reduces this from 45% to 23%.

**Fig 9:**
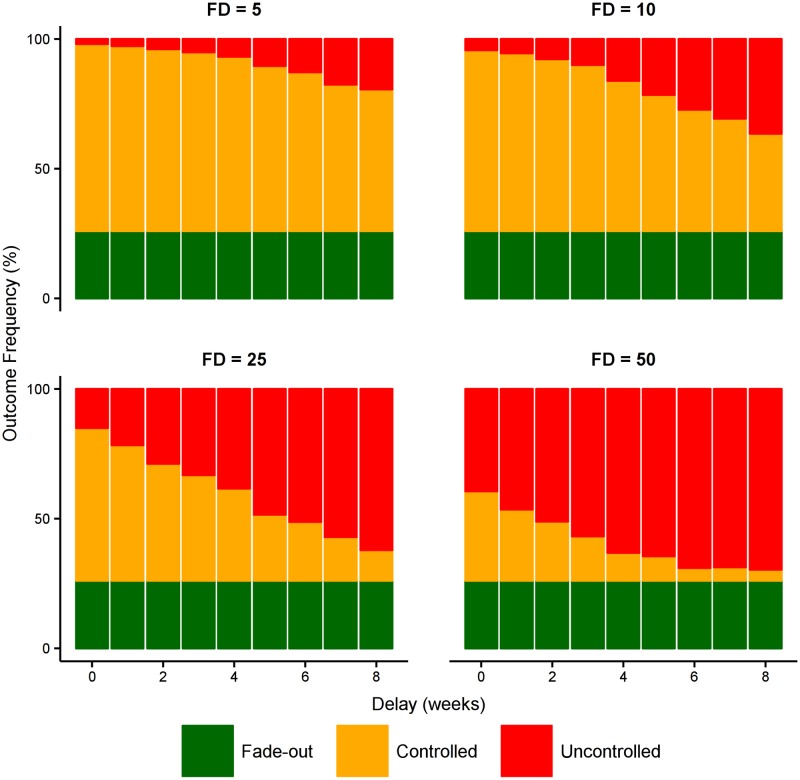
Effect of reducing force of infection from community transmission. Starting with a baseline case ascertainment of 70% and a small health care system (0.1% of population are HCWs, 1:15 and 1:50 ratio of bed and contact-tracing capacities to HCWs, as above), this figure shows the effect of reducing the force of infection from dead bodies (β_D_) and in the community (β_I_) by 25%. This represents a sociocultural intervention that changes both burial practices and social mixing in the community. Similar to Fig 7, success of such an intervention relies upon early detection and a rapid response from the community.

As expected, reducing the force of infection from both dead bodies and from infectious individuals in the community has a dramatic effect on disease transmission and substantially increases the likelihood of control (Fig 9). However, even the impact of this intervention is substantially reduced by late detection and delayed delivery.

### Results in context: Papua New Guinea

We now demonstrate how the above results can be interpreted with respect to a specific country in the Asia Pacific region. Papua New Guinea (PNG) was chosen as a suitable exemplar for several reasons. The Asia Pacific Strategy for Emerging Diseases (APSED) data demonstrated generally lower levels of capacity among Pacific Island countries [19]. Although PNG’s Human Development Index (HDI) (0.505 in 2014) is slightly higher than that of EVD-affected West African countries (0.411 in Guinea, 0.413 in Sierra Leone and 0.43 in Liberia), it is the lowest ranked country in the Southeast Asia/West Pacific region, and among the lowest ranked countries globally. Therefore, estimates of health care system and population factors relevant to disease transmission are likely to be comparable. The geographic proximity of PNG to Australia and Australia’s existing engagement in PNG make it likely that Australia would be called on to support the response to any outbreak in PNG [43].

First, we divided the country into high-level administrative regions (listed in Table 1) and used population sizes as reported in the 2011 census [44]. Estimates of hospital bed and health care worker numbers were provided by country experts at the Nossal Institute for Global Health (University of Melbourne, Australia), who suggested that no more than 10% of beds could be used for isolation of EVD cases. We also assumed a (modest) national contact-tracing capacity of 100 contacts per day. We allocated national hospital beds to Port Moresby and distributed remaining beds (regional and provincial hospitals) among the administrative regions in proportion to their population densities, as a proxy for both population size and health care system accessibility. The health care workforce and contact-tracing capacity were allocated among regions in proportion to their designated bed capacities. From these data, we estimate that the Port Moresby region has a medium health care system (almost 0.2% of the population are health care workers), that the Islands region has a small health care system (almost 0.1% are health care workers), and the remaining regions have even less health care infrastructure.

**Table 1:** The administrative regions of Papua New Guinea. We separate Port Moresby (the largest city and national capital) from the rest of the Southern (“Papua”) region on the grounds that Port Moresby comprises a much more urbanised population than the rest of the region.

Region	Population	Type	Isolation Beds	Contact Tracing	HCWs	% HCWs
Port Moresby	365,000	Urban	60	15	711	0.195%
Southern	1,091,250	Rural	24	6	279	0.026%
Highlands	2,854,874	Rural	193	50	2290	0.080%
Momase	1,867,657	Rural	57	15	672	0.036%
Islands	741,538	Rural	56	14	669	0.090%
***Total***			***390***	***100***	***4621***	

While Port Moresby is the largest city in Papua New Guinea and the commercial centre of the country, the vast majority of the population live elsewhere, and country experts at the Nossal Institute also advised that residents of Port Moresby frequently visit friends and relatives in rural regions upon return from international travel. Accordingly, it was judged pertinent to consider the probability of outbreak control in each of the regions. We assumed that the force of infection in the community was twice as high in Port Moresby (highly urbanised population) than in the other regions (with population densities 100 times lower than Port Moresby), and that the average time to burial following death was 3 days, allowing for the return of bodies to the home village for burial.

To estimate the case ascertainment proportion in Papua New Guinea, we consulted a variety of data sources and also referred back to the EVD outbreak in West Africa. Despite the high proportion of EVD cases that are symptomatic, early in the West African outbreak it was estimated that only about 40% of all cases sought clinical care [16]. Papua New Guinea has a low HDI ranking similar to those of the West African nations, and faces similar challenges in providing access to and quality of health care services. These challenges include: very few health professionals (less than 1 doctor per 10,000 people), insufficient health workforce in rural areas (more than 80% of medical officers work in urban areas, despite 87.5% of the population living in rural areas), low levels of workforce retention in rural areas (due to remoteness, financial instability, and dangerous environments), limited availability of basic essential medical supplies, and lack of access to clean water in many facilities [45]. Given these issues of access and provision, we assumed that case ascertainment in Papua New Guinea was unlikely to be higher than in the West African outbreak (40–50%) and, particularly in rural areas, might be substantially lower (10–20%).

With clear evidence of successful behavioural interventions in Papua New Guinea during the recent 2009 cholera outbreak as reported in [41], we anticipated that the force of infection in the community and from dead bodies could be substantially reduced once an EVD case was identified in the community. The geographic spread of the rural population (on the order of 10 people per square kilometre) and the inaccessibility of the Highlands region suggest that rapid delivery of additional isolation beds, health care workers, and enhanced contact tracing might be impractical outside of Port Moresby.

**Fig 10:**
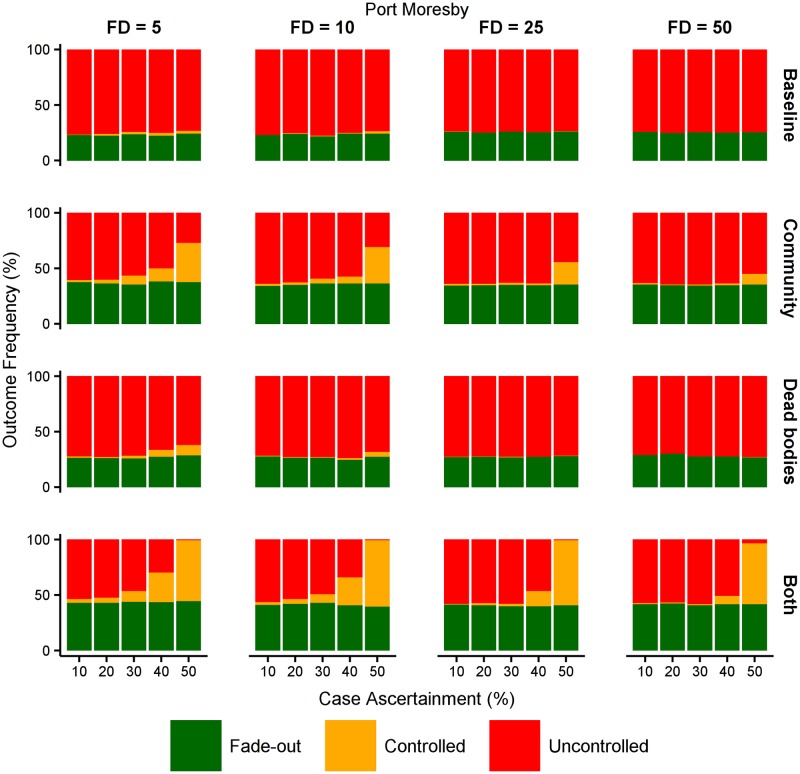
Effect of behavioural interventions in Port Moresby region. The effect of reducing the force of infection in the community and/or from dead bodies by 25%, in the urban population of Port Moresby where community transmission is high. When case ascertainment is 40% or higher, reducing transmission in both settings has a synergistic effect on outbreak control.

**Fig 11:**
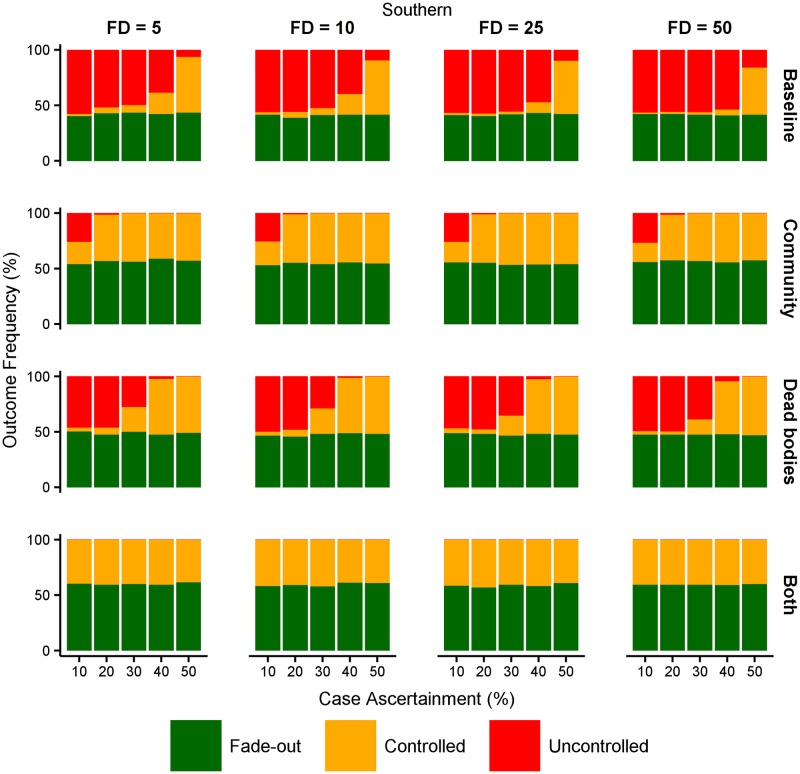
Effect of behavioural interventions in Southern region. The effect of reducing the force of infection in the community and/or from dead bodies by 25%, in the rural population of the Southern region where community transmission is low. When transmission is reduced in both settings, the overall force of infection is sufficiently low that uncontrolled outbreaks never occur.

In the densely populated urban setting of Port Moresby, controlled outbreaks are only likely under two sets of circumstances (see Fig 10). The first is when the first case is detected early, subsequent case ascertainment is high, and behavioural interventions reduce transmission in the community. The second is when case ascertainment is high and behavioural interventions reduce transmission in the community and also from dead bodies. In contrast, in the more sparsely populated rural settings, such as the Southern region, controlled outbreaks are likely when case ascertainment is high even in the absence of any behavioural interventions (see Fig 11). In these rural settings, behavioural interventions can greatly increase the likelihood of controlling outbreaks even when case ascertainment is very low, and the synergistic effects of reducing transmission in the community and also from dead bodies can avoid uncontrolled outbreaks altogether. We further considered interventions that delivered additional isolation beds, personal protective equipment, and health care workers, but the probability of outbreak control was not meaningfully affected, indicating that in these model scenarios the health care resources were not a limiting factor (see Other interventions in Papua New Guinea in S1 Methods).

## Discussion

Here we discuss our modelling results from the perspective of EVD in the Asia Pacific region, and more general lessons for mathematical modelling of EID outbreaks in data-poor settings.

### EVD in an Asia Pacific context

We have shown how the use of a stochastic model of infection and medical and social interventions can be used to support risk assessment and epidemic preparedness in the presence of limited data concerning the pathogen, the population, and available health care infrastructure. We have demonstrated how, over a range of likely country- and disease-specific scenarios, the probability of controlling an EVD outbreak is dependent on early detection and ongoing ascertainment. We have also shown that, in specific settings (such as the Papua New Guinea case study), the relative merits of different resourcing strategies may shift. For example, sensitivity analyses for the rural regions of PNG indicated that interventions targeting behavioural change, such as changes to burial practices, consistently improve controllability. However, providing extra hospital beds alone is unlikely to confer substantial benefits because the limiting factor is case ascertainment rather than treatment and isolation of cases. These observations are of course specific to this population and health care system context, and a strength of this framework is the ability to tailor intervention priorities to specific local context.

We can also make several more general observations. Changes in social mixing and burial practices, where the population is amenable to such interventions, can reduce transmission substantially and greatly increase the likelihood of control. This demonstrates the critical importance of effectively communicating infection control and hygiene measures to the community, and of establishing public trust in the health care system. Similarly, the provision of additional health care resources can increase the chance of control, but only if the existing infrastructure is the limiting factor in controlling an outbreak.

With regard to timing of interventions, social interventions and health care strengthening can be effective even when delivered several weeks after an outbreak has been detected, when the existing health care system is sufficient to accommodate patients in those early weeks. On the other hand, reactive strengthening of surveillance systems is likely to be ineffective: case ascertainment must be high from the start of the outbreak in order to effectively improve chances of control.

For future EVD outbreaks, vaccination is also likely to play a critical role in any response effort [46]. While we have not considered the effect of an EVD vaccine in this study, the model framework is amenable to such extension. Key questions to be addressed would then concern the ability of health agencies to obtain and distribute vaccines in an optimal fashion. In the event that a new infectious disease appears, however, vaccine availability cannot be expected for many months and effective responses will necessarily rely upon health care infrastructure and social interventions as modelled in this study.

The benefits of an effective surveillance system are broader than merely improving detection [47]. As well as enabling a more timely response to the West African EVD outbreak, an established surveillance system would have provided earlier and more accurate information on the number, location and characteristics of cases. It is plausible to reason that an established surveillance system could also play a role in communication with affected populations, improving efforts to modify social behaviour and increasing the trust of populations in health care systems.

It is instructive to compare our results with the qualitative risk assessment undertaken by WHO offices in September 2014, which focused on Pacific Island countries [14]. The assessment identified 3 scenarios:

1. A single case of EVD: due to relative low levels of travel between the Pacific and West Africa;
2. A localized cluster due to transmission in a health facility or social contacts. The assessment noted ‘If an imported case occurs, the chance of limited secondary transmission would be moderate to high. Frontline health workers, patients, health facility visitors and social contacts would likely be exposed’; and
3. Widespread and intense transmission. The assessment noted ‘Even if there is a localized cluster, the chance of progression to widespread and intense transmission of EVD in the Region would be very low, as there would need to be undetected cases of EVD in the community. As EVD is severe, most cases would present to a health facility for treatment’ [14].

Our mathematical modelling identified and clarified conditions for fade-out of a small cluster, and controlled and uncontrolled outbreaks. This clarifies the potential connection between what the WHO assessment refers to as ‘localized clusters’ and ‘widespread transmission’. While the WHO assessment identifies the need for clinician awareness, case definition, triage and precautions for cases, our model also highlights the importance of secondary contact tracing and case ascertainment in limiting further spread. Our modelling demonstrates that the risk of extending to widespread transmission is not negligible, and that the presentation of cases to health facilities would not in and of itself necessarily improve control. In agreement with the APSED strategy recommendations, our results highlight the critical importance of early detection and high ascertainment for EID control [19]. Preemptive strengthening of surveillance systems in this region is therefore of paramount importance.

A further use of mathematical models is to provide estimates of population and health care system impact that can serve as the basis for estimates of the economic impact of an EVD outbreak in the Asia-Pacific region [48]. Potential economic costs include not only the increased health costs and reduced labour productivity during the outbreak, but also the permanent reductions in a country’s population and labour force due to mortality, and behavioural effects induced by the outbreak such as reductions in international tourism and crowd-avoidance behaviour by a country’s residents. An estimate of these economic costs can inform the decisions that international agencies make about the benefits of intervention and health care system support.

There are several limitations to the generality of our results. As recognised above, health care system data is often of variable quality in many countries. In particular, such data are typically not available at a high spatial resolution, which is likely to hide considerable geographic heterogeneity in health care system quantity, quality and access. We have dealt with this limitation by exploring a range of potential health care system resourcing levels, but the availability of more accurate data would improve the quality of model scenarios. Furthermore, our modelling approach embodies particular assumptions about disease transmission characteristics that may only partially reflect the dynamics of any specific EID outbreak. For example, increased variability (‘overdispersion’) in the number of secondary cases that a primary case generates may lead to an overestimate in the risk of an outbreak occurring [37] (see Over-dispersion in secondary cases in S1 Methods).

### Lessons for EID modelling in data-poor settings

We can identify several general principles from this modelling study that can be used to inform decision making in data-poor settings.

A basic understanding of the pathogen is necessary to inform the model structure (i.e., to identify the disease-related compartments and the flows between them). Where disease transmission involves a separate vector (e.g., mosquitoes) or source (e.g., contaminated waterways), the model will require further compartments to capture these qualities. Model parameters may be informed by data from previous or related outbreaks, although such values should be validated as data for the novel pathogen become available.

In the absence of data beyond basic population demographics, expert local knowledge is critical for estimating available health care infrastructure, relevant socio-cultural behaviours that may influence disease transmission, and subsequent behavioural changes instigated by the perceived threat or by communications (e.g., community hygiene promotion). The inherent uncertainties in these factors highlights the importance of thorough sensitivity analyses, rather than relying on ‘best available’ point estimates [49].

Once the disease model has been defined and calibrated to the target population and health care system, the next challenge is to design a range of simulation scenarios that admit interventions and resourcing levels that are realistic in both scope and timeliness. Possible sources of guidance include previous outbreaks experienced by the target population, and previous foreign-aid efforts. For comparison against these scenarios, a set of simulations with no health care response should be generated to form a baseline.

Finally, it is important to determine how best to classify and communicate the epidemic outcomes generated by the model. Communication of key model outcomes to policy makers and local health care providers must be both accurate and comprehensible. Given the considerable uncertainty around model inputs in data-poor settings, it is important that specific quantitative outputs are not ‘over-interpreted’. For example, projections of cumulative cases are unlikely to be realised given both initial uncertainty, and the evolving nature of the local and international response and intervention. We therefore advocate focusing on qualitative measures of control and burden that can inform estimates of comparative, rather than absolute, impact. Our classification of outbreaks as either controlled or uncontrolled was appropriate in the case of EVD, since there was a clear division between epidemic sizes. It is possible that further distinctions may be required in other scenarios.

Despite the paucity of social, demographic and health care system data that is available for understudied countries, mathematical modelling can enable rapid assessment of risk across a range of country and intervention scenarios, to assist with prioritisation of EID preparedness and response efforts.

## Supporting Information

S1 Methods Model description, scenario parameters and additional results. (PDF)
