# Supplementary material for: Model-Informed Risk Assessment and Decision Making for an Emerging Infectious Disease in the Asia-Pacific Region
Source: PLoS Negl Trop Dis. 2016 Sep 23;10(9):e0005018. doi: 10.1371/journal.pntd.0005018 (PMC5035030; doi:10.1371/journal.pntd.0005018)
Supplement: S1 Methods — (PDF) [file pntd.0005018.s001.pdf]

Supplementary Information

Model-informed risk assessment and decision making for an emerging infectious disease in the Asia-Pacific region

Robert Moss, Roslyn I Hickson, Jodie McVernon, James M McCaw, Krishna Hort, Jim Black, John R Madden, Nhi H Tran, Emma S McBryde, Nicholas Geard

Model description

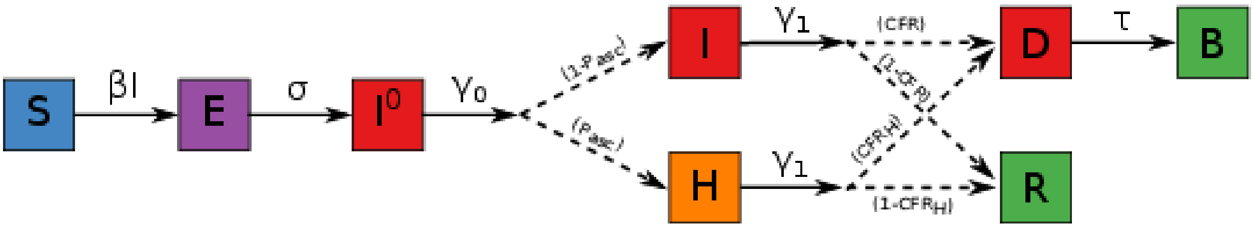

Figure 1: Model diagram, showing flows between compartments that represent the natural history of EVD and health care interventions.

We used a stochastic SEIR-type model (Figure 1), with the addition of compartments for the number of unburied dead bodies (which form a significant source of transmission, if there are interactions with the dead body, as is common in various funeral rites) and the number of safely buried dead bodies (assumed to no longer contribute to transmission). All individuals are initially susceptible to infection (S). Once infected, they enter a latent incubation stage (E), from which they transition to become infectious, but not yet symptomatic (I_0). At the point of developing symptoms (I), their infectiousness increases. Post-infection, individuals either recover and are no longer infectious (R) or die and remain infectious (D) until buried (B).

Tables 1 and 2 define, respectively, the states and parameters (together with base values) used in our model of Ebola virus disease transmission. Equations 1–30 specify the *deterministic* disease transmission model, which were used to define the transition probabilities for the discrete-time Markov chain model that we used to obtain stochastic outbreak realisations. Table 3 defines time-varying quantities that appear in the model equations.

State	Meaning
$S_C(t)$	Susceptible individuals in the general community
$S_T(t)$	Susceptible individuals monitored via contact tracing
$S_H(t)$	Susceptible health care workers
$E_C(t)$	Exposed individuals in the general community
$E_T(t)$	Exposed individuals monitored via contact tracing
$E_H(t)$	Exposed health care workers
$I_{0,C}(t)$	Infectious (early-stage) individuals in the general community
$I_{0,T}(t)$	Infectious (early-stage) individuals monitored via contact tracing
$I_{0,H}(t)$	Infectious (early-stage) health care workers
$H_C(t)$	Hospitalised individuals
$H_H(t)$	Hospitalised health care workers
$I_C(t)$	Infectious individuals in the general community
$I_H(t)$	Infectious health care workers
$D_C(t)$	Dead individuals
$D_H(t)$	Dead health care workers
$R_C(t)$	Recovered individuals
$R_H(t)$	Recovered health care workers
$B_C(t)$	Buried individuals
$B_H(t)$	Buried health care workers

Table 1: Model state definitions.

Symbol	Value	Meaning
N	10^5	Population size
F_{HCW}	0.001—0.002—0.003	Fraction of the population that are health care workers
e_0	1	The number of initial exposures
FD	*varies*	The number of infections that occur before the first case is identified
κ	20	Number of contacts per identified case
κ_I	1	Number of infected cases found per identified case
$N_{\mathrm{CT}}^{\max}$	1 per 50 HCWs	Contact tracing capacity
$N_{\mathrm{H}}^{\max}$	1 per 15 HCWs	Isolation ward bed capacity
τ_{CT}	$1/20$	Contact monitoring period of 20 days
σ	$1/10$	Incubation period of 10 days
γ_0	$1/4$	Pre-detection period of 4 days
γ_1	$1/6$	Post-detection period of 6 days
τ_B	$1/3$	Pre-burial period of 3 days
$\beta_{I,0}^{C}$	0.1	Force of infection (community) from early-stage infectiousness
$\beta_{I,0}^{H}$	0.1	Force of infection (health care) from early-stage infectiousness
$\beta_{I,C}^{C}$	0.2	Force of infection (community) from infectious cases in the community
$\beta_{I,C}^{H}$	0.3—0.2	Force of infection (health care) from infectiousness cases in the community
β_{H}^{C}	0	Force of infection (community) from hospitalised cases
β_{H}^{H}	0.02	Force of infection (health care) from hospitalised cases
β_{D}^{C}	0.2	Force of infection (community) from dead, unburied cases
β_{D}^{H}	0.2	Force of infection (health care) from dead, unburied cases
CFR_I	0.7	Case fatality ratio for individuals outside of health care settings
CFR_H	0.35	Case fatality ratio for individuals in hospital

Table 2: Model parameter definitions.

Symbol	Meaning
$p_{\mathrm{asc}}(t)$	The probability of ascertaining a newly-symptomatic case
$\beta_C(t)$	Force of infection experienced in the community
$\beta_H(t)$	Force of infection experienced by health care workers
$N_{\mathrm{CT}}(t)$	The number of individuals that become subject to contact tracing
$N_{\mathrm{asc}}(t)$	The number of ascertained cases
$p_E(t)$	The number of traced cases that are drawn from E_C and not $I_{0,\mathrm{C}}$
$F_{\mathrm{H,H}}(t)$	Fraction of infectious health care workers sent to hospital
$F_{\mathrm{C,H}}(t)$	Fraction of infectious community cases sent to hospital
$\epsilon(t)$	Healthcare capacity, as a function of available health care workers

Table 3: Time-varying quantities in the model.

$$S_C(0) = N - S_H(0) - E_C(0) \tag{1}$$
$$S_H(0) = [N \cdot F_{\text{HCW}}] \tag{2}$$
$$E_C(0) = e_0 \tag{3}$$
$$\frac{dS_C}{dt} = -\beta_C(t) \cdot \frac{S_C}{N} - \frac{\kappa - \kappa_I}{\kappa} \cdot N_{\text{CT}} + S_T \cdot \tau_{\text{CT}} \tag{4}$$
$$\frac{dS_T}{dt} = -\beta_C(t) \cdot \frac{S_T}{N} + \frac{\kappa - \kappa_I}{\kappa} \cdot N_{\text{CT}} - S_T \cdot \tau_{\text{CT}} \tag{5}$$
$$\frac{dS_H}{dt} = -\beta_H(t) \cdot \frac{S_H}{N} \tag{6}$$
$$\frac{dE_C}{dt} = \beta_C(t) \cdot \frac{S_C}{N} - p_E \cdot \frac{\kappa_I}{\kappa} \cdot N_{\text{CT}} - \sigma \cdot E_C \tag{7}$$
$$\frac{dE_T}{dt} = \beta_C(t) \cdot \frac{S_T}{N} + p_E \cdot \frac{\kappa_I}{\kappa} \cdot N_{\text{CT}} - \sigma \cdot E_T \tag{8}$$
$$\frac{dE_H}{dt} = \beta_H(t) \cdot \frac{S_H}{N} - \sigma \cdot E_H \tag{9}$$
$$\frac{dI_{0,\text{C}}}{dt} = \sigma \cdot E_C - (1 - p_E) \cdot \frac{\kappa_I}{\kappa} \cdot N_{\text{CT}} - \gamma_0 \cdot I_{0,\text{C}} \tag{10}$$
$$\frac{dI_{0,\text{T}}}{dt} = \sigma \cdot E_T + (1 - p_E) \cdot \frac{\kappa_I}{\kappa} \cdot N_{\text{CT}} - \gamma_0 \cdot I_{0,\text{T}} \tag{11}$$
$$\frac{dI_{0,\text{H}}}{dt} = \sigma \cdot E_H - \gamma_0 \cdot I_{0,\text{H}} \tag{12}$$
$$\frac{dI_C}{dt} = (1 - p_{\text{asc}}(t)) \cdot \gamma_0 \cdot I_{0,\text{C}} + (1 - F_{C,H}) \cdot p_{\text{asc}}(t) \cdot \gamma_0 \cdot I_{0,\text{C}} - \gamma_1 \cdot I_C \tag{13}$$
$$\frac{dI_H}{dt} = (1 - F_{H,H}) \cdot \gamma_0 \cdot I_{0,\text{H}} - \gamma_1 \cdot I_H \tag{14}$$
$$\frac{dH_C}{dt} = F_{C,H} \cdot p_{\text{asc}}(t) \cdot \gamma_0 \cdot I_{0,\text{C}} - \gamma_1 \cdot H_C \tag{15}$$
$$\frac{dH_H}{dt} = F_{H,H} \cdot \gamma_0 \cdot I_{0,\text{H}} - \gamma_1 \cdot H_H \tag{16}$$
$$\frac{dD_C}{dt} = \text{CFR}_I \cdot \gamma_1 \cdot I_C + \text{CFR}_H \cdot \gamma_1 \cdot H_C \tag{17}$$
$$\frac{dD_H}{dt} = \text{CFR}_I \cdot \gamma_1 \cdot I_H + \text{CFR}_H \cdot \gamma_1 \cdot H_H \tag{18}$$
$$\frac{dR_C}{dt} = (1 - \text{CFR}_I) \cdot \gamma_1 \cdot I_C + (1 - \text{CFR}_H) \cdot \gamma_1 \cdot H_C \tag{19}$$
$$\frac{dR_H}{dt} = (1 - \text{CFR}_I) \cdot \gamma_1 \cdot I_H + (1 - \text{CFR}_H) \cdot \gamma_1 \cdot H_H \tag{20}$$
$$\frac{dB_C}{dt} = D_C \cdot \tau_B \tag{21}$$
$$\frac{dB_H}{dt} = D_H \cdot \tau_B \tag{22}$$

All compartments are initially set to zero, except for the susceptible community and health care worker populations, and the initially-exposed individuals (Equations 1–3). The deterministic equations (4–22) define the transition probabilities for the discrete-time stochastic infection model that was used to generate the results presented in the accompanying manuscript.

$$\beta_C(t) = \beta_{\text{I},0}^{C} \cdot (I_{0,\text{C}} + I_{0,\text{H}} + I_{0,\text{T}}) + \beta_{\text{I,C}}^{C} \cdot (I_C + I_H) + \beta_H^C \cdot (H_C + H_H) + \beta_D^C \cdot (D_C + D_H) \quad (23)$$

$$\beta_H(t) = \beta_{\text{I},0}^{H} \cdot (I_{0,\text{C}} + I_{0,\text{H}} + I_{0,\text{T}}) + \beta_{\text{I,C}}^{H} \cdot (I_C + I_H) + \beta_H^H \cdot (H_C + H_H) + \beta_D^H \cdot (D_C + D_H) \quad (24)$$

$$N_{\text{CT}}(t) = \min\left(N_{\text{asc}} \cdot \kappa, N_{\text{CT}}^{\max} \cdot \epsilon - S_T - E_T - I_{0,\text{T}}, 0\right) \quad (25)$$

$$N_{\text{asc}}(t) = F_{C,H} \cdot p_{\text{asc}}(t) \cdot \gamma_0 \cdot I_{0,\text{C}} \quad (26)$$

$$p_E(t) = \frac{E_C}{E_C + I_{0,\text{C}}} \quad (27)$$

$$F_{\text{H,H}}(t) = \frac{N_{\text{H}}^{\max} \cdot \epsilon - H_C - H_H}{\gamma_0 \cdot I_{0,\text{H}}} \in [0,1] \quad (28)$$

$$F_{\text{C,H}}(t) = \frac{N_{\text{H}}^{\max} \cdot \epsilon - H_C - H_H - \gamma_0 \cdot I_{0,\text{H}}}{p_{\text{asc}}(t) \cdot \gamma_0 \cdot I_{0,\text{C}}} \in [0,1] \quad (29)$$

$$\epsilon(t) = \text{Beta}_{\text{PPF}}\left(\alpha = 0.01, \beta = 0.01, x = \frac{S_H + R_H}{S_H(0)} - 0.4\right) \quad (30)$$

The force of infection in the community (Equation 23) and in the health care workforce (Equation 24) are time-varying quantities that depend on disease prevalence. The number of individuals that have become subject to contact tracing at each time step (Equation 25) depends on the available contact tracing capacity (subject to the effective health care capacity ϵ and the number of contacts already being traced) and on the number of cases that ascertained in that time step (Equation 26). Infected contacts, identified at a rate κ_I per identified case, are drawn from E_C and $I_{0,\text{C}}$ in proportion to the number of people in these two states (Equation 27).

The fraction of health care workers that are hospitalised and enter H_H rather than I_H (Equation 28) depends upon the *effective* number of beds and the number of occupied beds (H_H and H_C). The fraction of community cases that are hospitalised and enter H_C rather than I_C (Equation 29) depends upon the *effective* number of beds and the number of occupied beds *including* health care workers that were hospitalised during this time step.

The *effective* health care capacity, expressed as a fraction ϵ of the actual capacity, is a function of the available health care workforce (Equation 30) and is illustrated in Figure 2. This equation smoothly decreases health care capacity due to infection of health care workers, both as a direct consequence of infection and, for uninfected health care workers, due to perceived risk of infection as a consequence of providing health care services. This relationship is defined using the quantile (percent point) function for the Beta distribution (Beta_{PPF}, for $\alpha = \beta = 0.01$), chosen solely on the grounds that it has the desired shape.

The case ascertainment probability $p_{\text{asc}}(t)$ was kept fixed at 0 until a pre-defined number of individuals FD became symptomatic (i.e., transitioned to I_C, I_H, or H_H), at which point the first detected case was defined have occurred and $p_{\text{asc}}(t)$ became non-zero (as defined in the simulation scenario tables, below).

Numerical simulation of stochastic model

At each time-step, the outflow from each compartment is sampled from a Poisson distribution whose mean value is the deterministic outflow rate (as defined by the equations below). Where these outflows are delivered to multiple destination compartments subject to a probability (e.g., the ascertainment of cases leaving $I_{0,\text{C}}$ with probability p_{asc}), the division of the outflow is sampled from a binomial distribution. Where an outflow is delivered to multiple destination compartments subject to capacity constraints (e.g., the hospitalisation and home-based quarantine of ascertained cases leaving $I_{0,\text{C}}$), priority is determined by strata; health-care workers receive the highest priority, followed by monitored cases, followed by cases in the general community. The remaining outflows, if any, are sent elsewhere (e.g., the overflow of ascertained cases from $I_{0,\text{C}}$ is delivered to I_C, and the overflow of cases from $I_{0,\text{H}}$ is delivered to I_H).

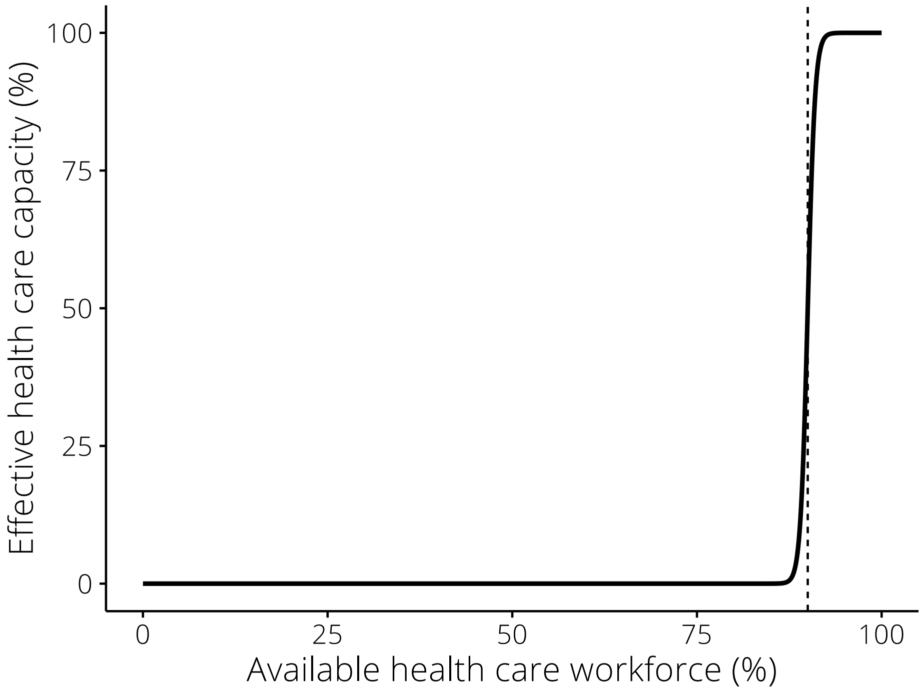

Figure 2: The effective health care capacity ϵ is a function of the available health care workforce (i.e., those in S_H and R_H, who have either avoided infection or who have recovered from infection). Effective capacity is decreased by 50% when 10% of health care workers are infected (vertical dashed line) and rapidly decreases to 0% (i.e., complete absence of health care services).

Simulation scenarios

Case ascertainment and health care capacity

Symbol	Value(s)
p_{asc}	$0^{\dagger}$ — 10%, 20%, ..., 100%
F_{HCW}	0.001, 0.002, 0.003
$N_{\text{CT}}^{\text{max}}$	1 per 50 HCWs
$N_{\text{H}}^{\text{max}}$	1 per 15 HCWs
FD	5, 10, 25, 50

Table 4: Simulation parameters used to explore the epidemic outcomes as a function of case ascertainment, subject to the first detected case and the health care system capacity.
†: No cases are ascertained until FD cases have occurred (i.e., have transitioned to I_C, I_H, or H_H).

For each combination of parameters listed in Table 4, we simulated 1,000 model realisations.

Increasing case ascertainment

Symbol	Value(s)
p_{asc}	$0^{\dagger}$ — $20\%^{\ddagger}$ — 20% + B
B	10%, 20%, ..., 80%
D	0, 7, 14, ..., 56
F_{HCW}	0.001
FD	5, 10, 25, 50

Table 5: Simulation parameters used to explore the epidemic outcomes as a function of delayed boosts to the case ascertainment probability.
†: No cases are ascertained until FD cases have occurred (i.e., have transitioned to I_C, I_H, or H_H).
‡: Case ascertainment remains fixed at 20% until D days after the ascertainment of the first case, and is then boosted by B%.

For each combination of parameters listed in Table 5, we simulated 1,000 model realisations.

Increasing health care capacity

Symbol	Value(s)
p_{asc}	$0^{\dagger}$ — 80%
B	0.1, 0.25, 0.5, 1.0
D	0, 7, 14, ..., 56
F_{HCW}	0.001
FD	5, 10, 25, 50
CT_0	$N/15 \cdot F_{\text{HCW}}$
H_0	$N/50 \cdot F_{\text{HCW}}$
$N_{\text{CT}}^{\max}$	$\text{CT}_0^{\ddagger}$ — $\text{CT}_0 \cdot (1+B)$
$N_{\text{H}}^{\max}$	$\text{H}_0^{\ddagger}$ — $\text{H}_0 \cdot (1+B)$

Table 6: Simulation parameters used to explore the epidemic outcomes as a function of delayed boosts to the health care system capacity.
†: No cases are ascertained until FD cases have occurred (i.e., have transitioned to I_C, I_H, or H_H).
‡: Contact tracing and isolation bed capacities remain at their initial values until D days after the ascertainment of the first case, and are then boosted by B.

For each combination of parameters listed in Table 6, we simulated 1,000 model realisations.

Reducing the force of infection from dead bodies

Symbol	Value(s)
p_{asc}	$0^{\dagger}$ — 70%
R	10%, 25%, 50%, 75%, 90%
D	0, 7, 14, ..., 56
β_D^C	$0.2^{\ddagger}$ — $0.2 \cdot (1-R)$
β_D^H	$0.2^{\ddagger}$ — $0.2 \cdot (1-R)$
F_{HCW}	0.001
FD	5, 10, 25, 50

Table 7: Simulation parameters used to explore the epidemic outcomes as a function of delayed decreases in the force of infection from dead bodies.
†: No cases are ascertained until FD cases have occurred (i.e., have transitioned to I_C, I_H, or H_H).
‡: The force of infection from dead bodies remains fixed until D days after the ascertainment of the first case, and is then reduced by R%.

For each combination of parameters listed in Table 7, we simulated 1,000 model realisations.

Reducing the force of infection from dead bodies and in the community

Symbol	Value(s)
p_{asc}	$0^{\dagger}$ — 70%
R	10%, 25%, 50%, 75%, 90%
D	0, 7, 14, ..., 56
β_D^C	$0.2^{\ddagger}$ — $0.2 \cdot (1 - R)$
β_D^H	$0.2^{\ddagger}$ — $0.2 \cdot (1 - R)$
$\beta_{I,0}^C$	$0.1^{\ddagger}$ — $0.1 \cdot (1 - R)$
$\beta_{I,0}^H$	$0.1^{\ddagger}$ — $0.1 \cdot (1 - R)$
$\beta_{I,C}^C$	$0.2^{\ddagger}$ — $0.2 \cdot (1 - R)$
$\beta_{I,C}^H$	$0.3^{\ddagger}$ — $0.3 \cdot (1 - R)$
F_{HCW}	0.001
FD	5, 10, 25, 50

Table 8: Simulation parameters used to explore the epidemic outcomes as a function of delayed decreases in the forces of infection from dead bodies and from cases in the community.
†: No cases are ascertained until FD cases have occurred (i.e., have transitioned to I_C, I_H, or H_H).
‡: The forces of infection from dead bodies and from cases in the community remain fixed until D days after the ascertainment of the first case, and are then reduced by R%.

For each combination of parameters listed in Table 8, we simulated 1,000 model realisations.

Results in context: Papua New Guinea

Symbol	Values(s)	
p_{asc}	$0^{\dagger}$ — 10%, 20%, ..., 50%	
FD	5, 10, 25, 50	
R	25%	
β_D^C	$0.2^{\ddagger}$ — $0.2 \cdot (1 - R)$	
β_D^H	$0.2^{\ddagger}$ — $0.2 \cdot (1 - R)$	
$\beta_{I,0}^C$	$0.1^{\ddagger}$ — $0.1 \cdot (1 - R)$	
$\beta_{I,0}^H$	$0.1^{\ddagger}$ — $0.1 \cdot (1 - R)$	
$\beta_{I,C}^H$	$0.3^{\ddagger}$ — $0.3 \cdot (1 - R)$	
Symbol	Port Moresby	Rural regions
N	365,000	*varies*
F_{HCW}	0.00195	*varies*
$\beta_{I,C}^C$	$0.2^{\ddagger}$ — $0.2 \cdot (1 - R)$	$0.1^{\ddagger}$ — $0.1 \cdot (1 - R)$

Table 9: Simulation parameters used to explore the epidemic outcomes as a function of delayed decreases in the forces of infection from dead bodies and from cases in the community.
†: No cases are ascertained until FD cases have occurred (i.e., have transitioned to I_C, I_H, or H_H).
‡: The forces of infection are reduced by R% when the first case is ascertained.

Region	Population	Isolation Beds	Contacts	HCWs	F_{HCW}
Port Moresby	365,000	60	15	711	0.195%
Southern	1,091,250	24	6	279	0.026%
Highlands	2,854,874	193	50	2290	0.080%
Momase	1,867,657	57	15	672	0.036%
Islands	741,538	56	14	669	0.090%

Table 10: The administrative regions of Papua New Guinea, except that we separate Port Moresby (the largest city and national capital) from the rest of the Southern ("Papua") region on the grounds that Port Moresby comprises a much more urbanised population than the rest of the region.

For each combination of parameters listed in Table 9, and for each of the administrative regions listed in Table 10, we simulated 1,000 model realisations.

Other interventions in Papua New Guinea

We also explored the effect of international interventions that supported local health care systems by delivering additional isolation beds and health care workers. Two different intervention scenarios were considered:

- A rapid but small intervention that delivers an additional 100 beds and 100 health care workers two weeks after the first case is detected ("Small"); and
- A slower but larger intervention that delivers an additional 1,000 beds and 1,000 health care workers eight weeks after the first case is detected ("Large").

These two interventions were compared against the baseline scenario where no additional health care resources were made available ("None"). The results for each of the administrative regions (Table 10) are shown in Figures 3–7. In summary, while these interventions provide modest reductions in the frequency of uncontrolled outbreaks in some scenarios, they confer limited benefit compared to the behavioural interventions (reduction in transmission associated with community contacts and dead bodies) reported in the manuscript.

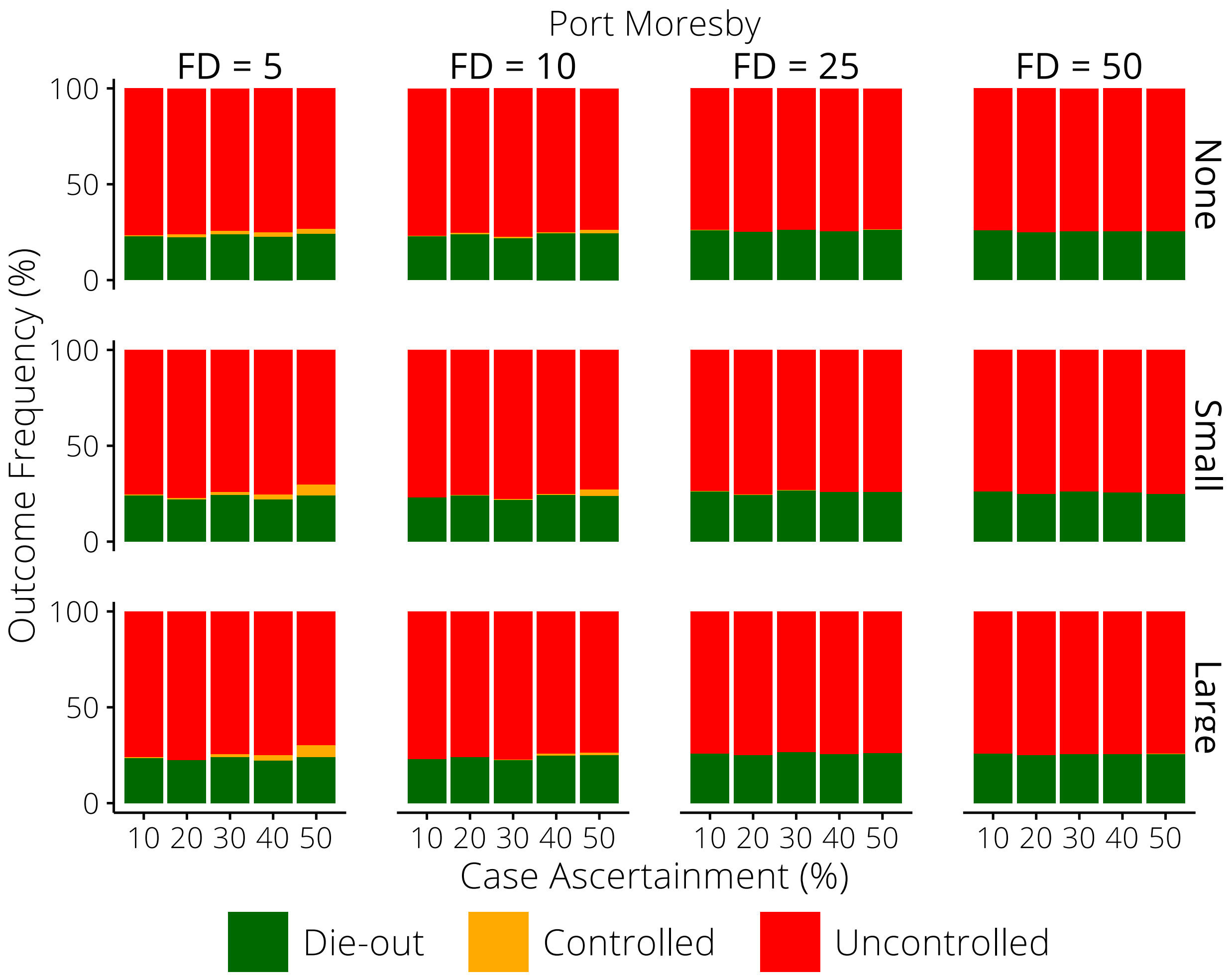

Figure 3: **Effect of delivery additional health care resources in Port Moresby region.** A minor decrease in the frequency of uncontrolled outbreaks can be observed when the first case is detected very early (e.g., "FD = 5") and case ascertainment is high (e.g., 50%).

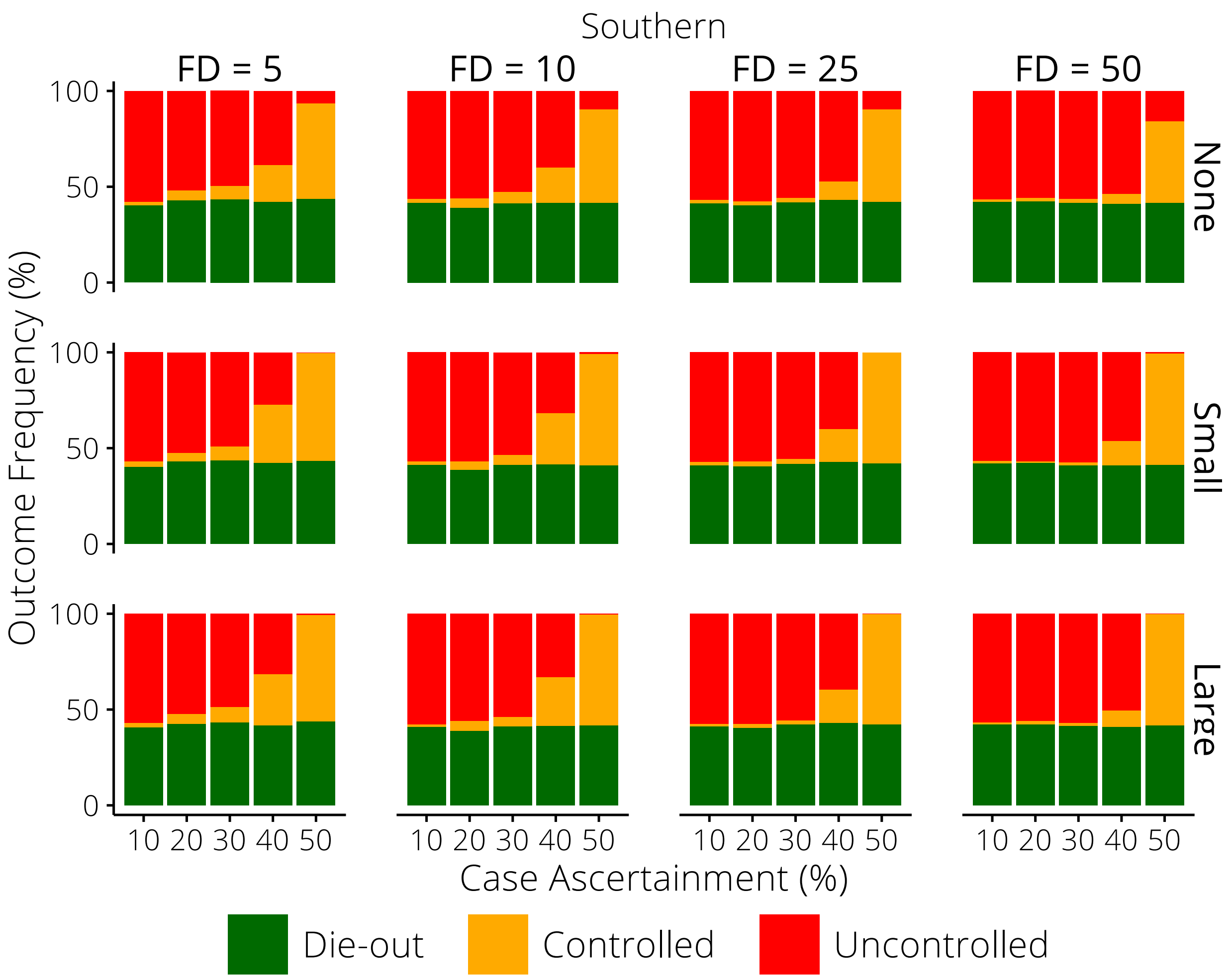

Figure 4: **Effect of delivery additional health care resources in Southern region.** In the absence of any intervention ("None"), uncontrolled outbreaks rarely occur if case ascertainment is 50%; the interventions are able to prevent uncontrolled outbreaks in these circumstances. The interventions provide little benefit when case ascertainment is 40%, and provide negligible benefit when case ascertainment is 30% or lower.

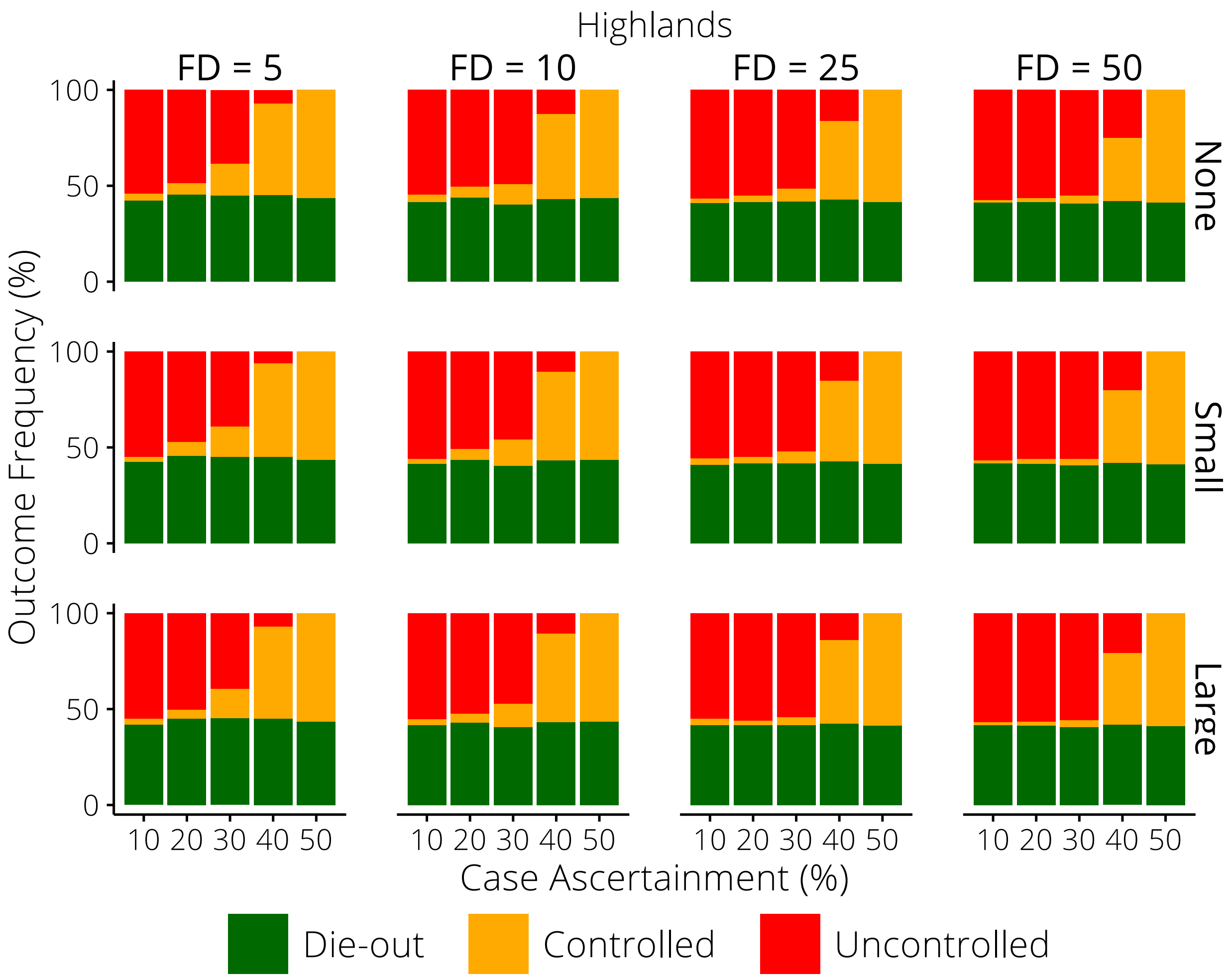

Figure 5: **Effect of delivery additional health care resources in Highlands region.** The interventions provide negligible benefit in all scenarios.

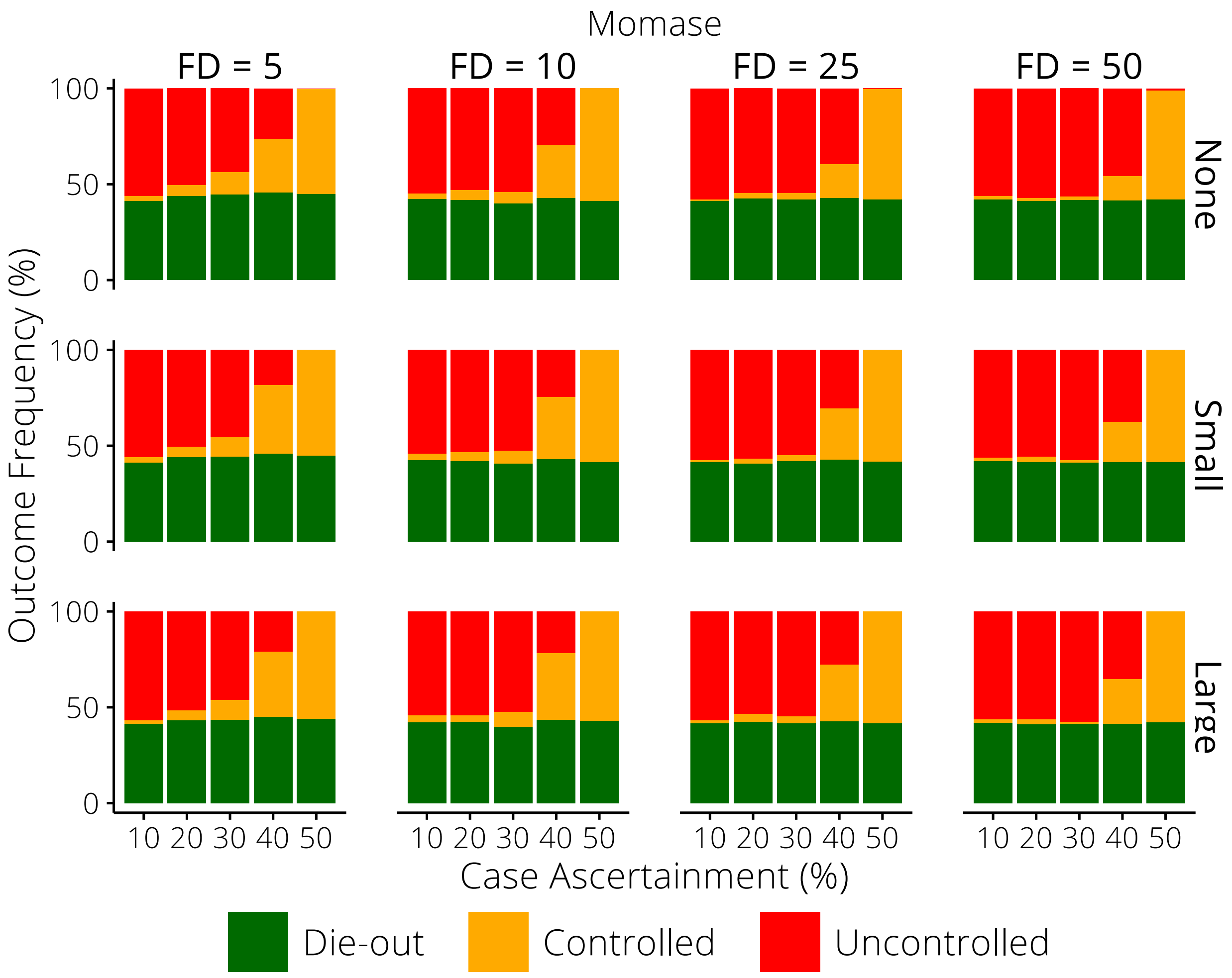

Figure 6: **Effect of delivery additional health care resources in Momase region.** The interventions provide little benefit when case ascertainment is 40%, and provide negligible benefit when case ascertainment is 30% or lower.

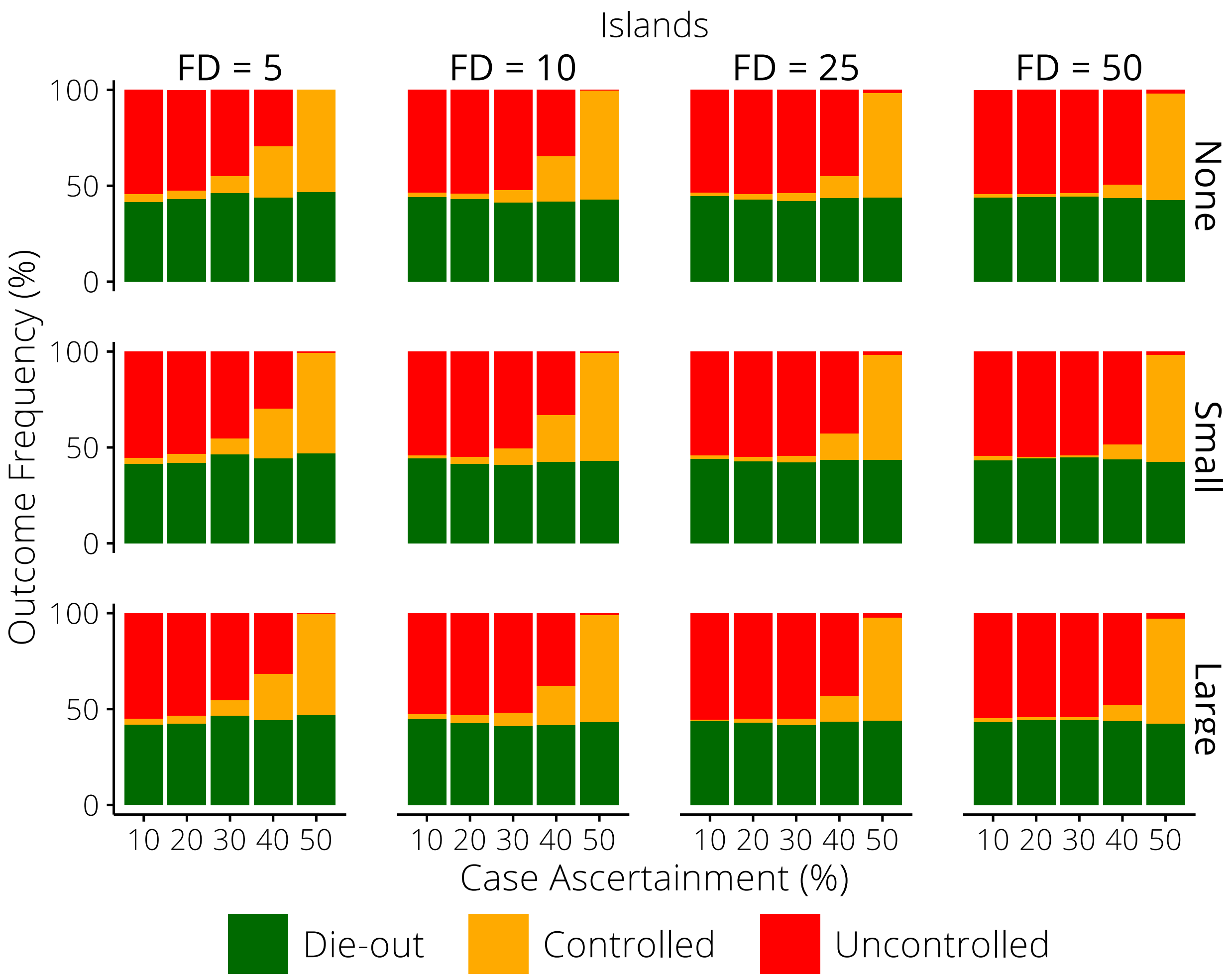

Figure 7: **Effect of delivery additional health care resources in Islands region.** The interventions provide negligible benefit in all scenarios.

Over-dispersion in secondary cases

By sampling the number of secondary cases from a Poisson distribution, the model presented here may under-estimate the actual variation in the number of secondary cases [1]. We can instead sample the number of secondary cases using probability distributions with means defined by the deterministic model equations (presented above) but which are over-dispersed with respect to the Poisson. The negative binomial distribution is frequently used in ecology and epidemiology, because it allows the mean and variance to be controlled independently.

The *dispersion parameter* k controls the variance: as k increases the variance decreases and the distribution approaches the Poisson (see Figure 8). When the number of secondary cases is highly dispersed (given a fixed mean), the likelihood of fade-out is increased, and uncontrolled outbreaks are observed less frequently and may have greater variation in their final size.

Toth et al. fitted negative binomial distributions to transmission data from the West Africa EVD outbreak as of April 24, 2015 ($n = 56$) and estimated the reproductive number R and dispersion parameter k for different patient subgroups [2]. Estimates of k produced wide confidence intervals, from values as low as 0.03 to larger values (> 1) consistent with the Poisson, and the authors selected three values of k for analysis: 0.1, 1, 10. We re-ran the model simulations for the urban population of Port Moresby, where the force of infection was reduced in the community and/or from dead bodies for these same values: $k = 10$ (Figure 10), $k = 1$ (Figure 11) and $k = 0.1$ (Figure 12). The results produced by the original model are shown in Figure 9.

The results for $k = 10$ and $k = 1$ do not differ substantially from those produced by the original model, although the proportion of simulations that result in fade-out or controlled epidemics is slightly higher when $k = 1$ than when $k = 10$. When $k = 0.1$ the likelihood of fade-out is higher in all scenarios than is observed for larger values of k. There is also a much greater chance of controlled epidemics, even in the absence of any reduction in the force of infection (i.e., the "Baseline"). However, the relative effects of case ascertainment and interventions are still observed.

References

[1] J. O. Lloyd-Smith, S. J. Schreiber, P. E. Kopp, and W. M. Getz. Superspreading and the effect of individual variation on disease emergence. *Nature*, 438(7066):355–359, Nov 2005. ISSN 1476-4687, `doi:10.1038/nature04153`.

[2] Damon J.A. Toth, Adi V. Gundlapalli, Karim Khader, Warren B.P. Pettey, Michael A. Rubin, Frederick R. Adler, and Matthew H. Samore. Estimates of outbreak risk from new introductions of Ebola with immediate and delayed transmission control. *Emerging Infectious Diseases*, 21(8):1402–1408, Aug 2015. ISSN 1080-6059, `doi:10.3201/eid2108.150170`.

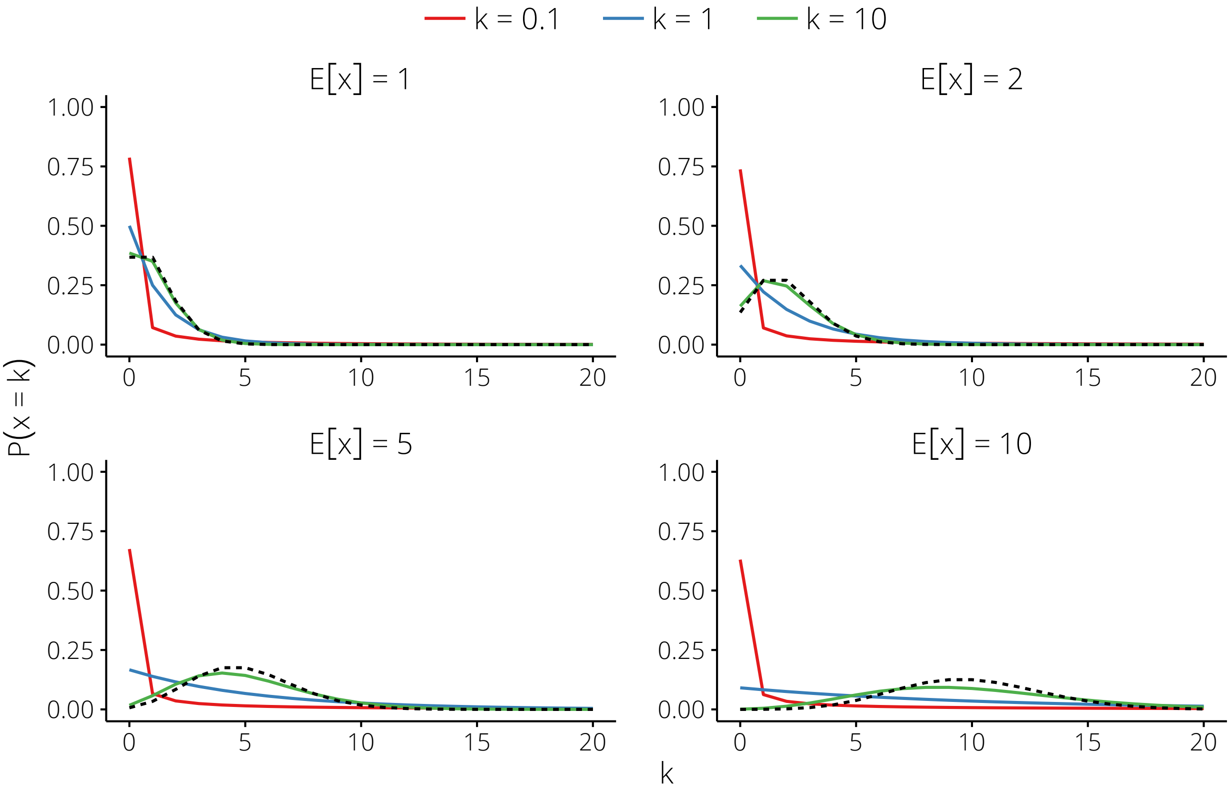

Figure 8: **Distribution of the number of secondary cases, for different values of k.** Negative binomial distributions, shown for different *expected* numbers of secondary cases ($E[x]$) and compared to the Poisson (dashed black line).

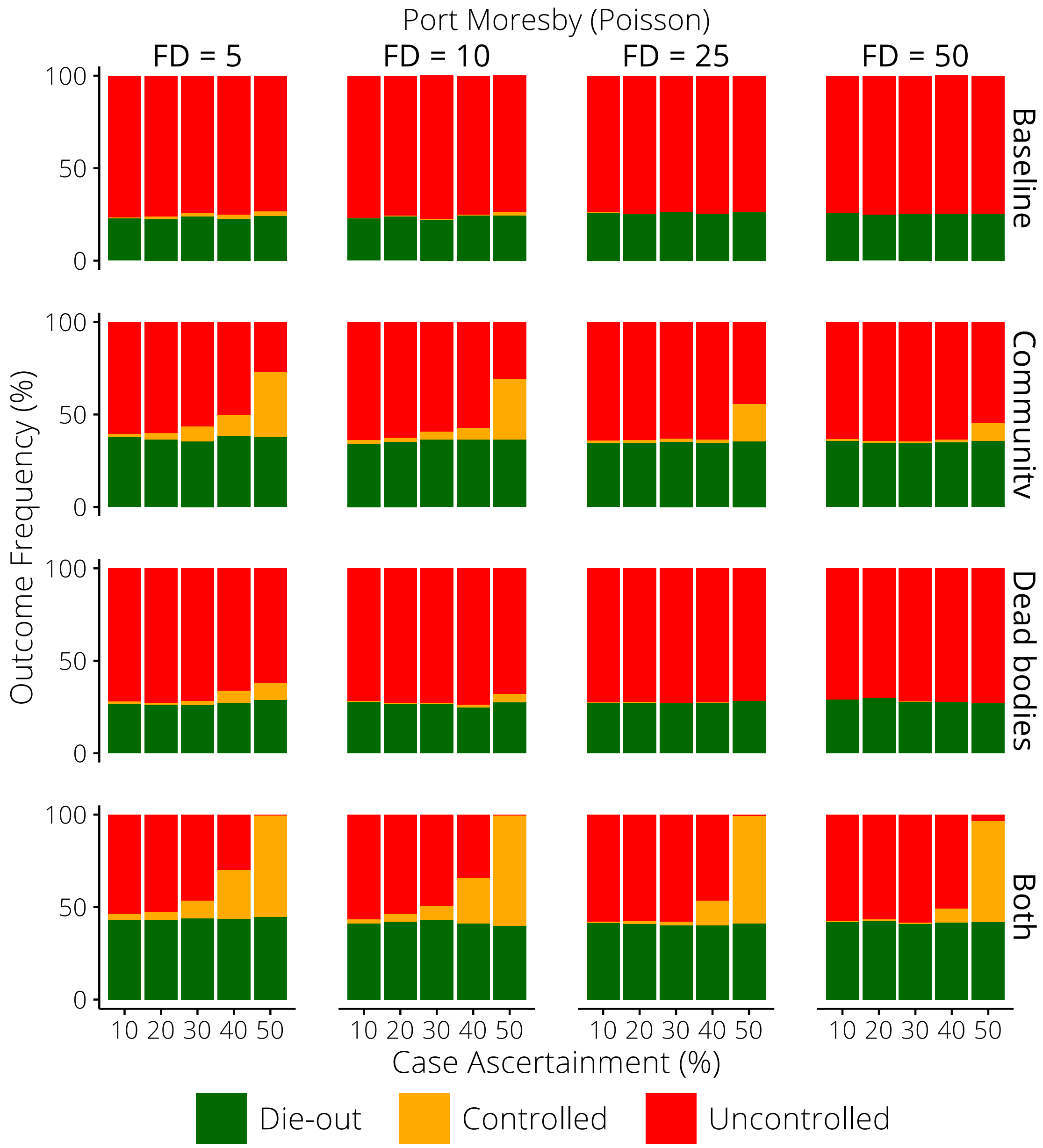

Figure 9: **Effect of behavioural interventions in Port Moresby region for the Poission.** The effect of reducing the force of infection in the community and/or from dead bodies by 25%, in the urban population of Port Moresby where community transmission is high, when the number of secondary cases are sampled from the Poisson (identical to Figure 10 of the manuscript).

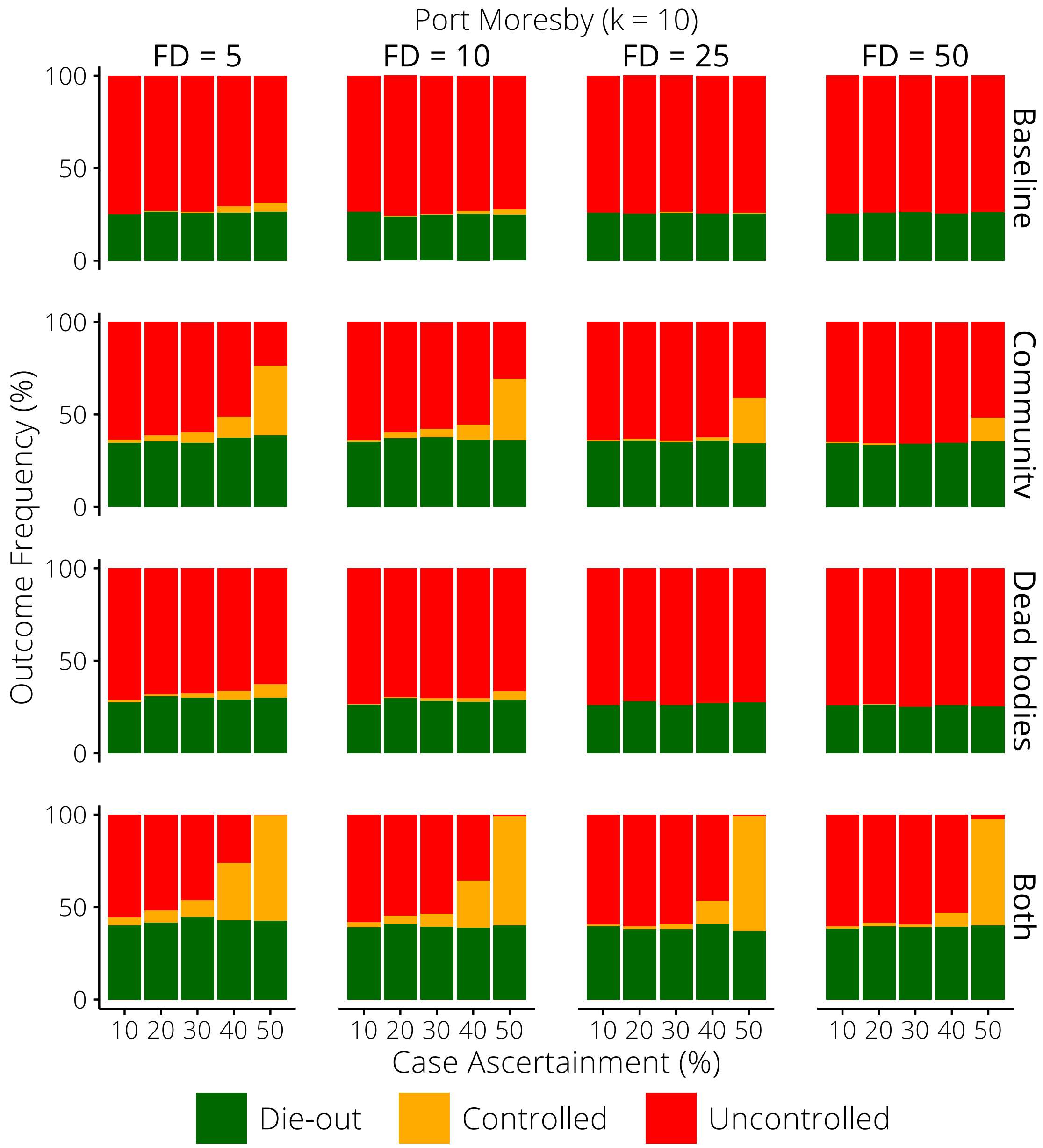

Figure 10: **Effect of behavioural interventions in Port Moresby region for $k = 10$.** The effect of reducing the force of infection in the community and/or from dead bodies by 25%, in the urban population of Port Moresby where community transmission is high, when the number of secondary cases are over-dispersed with respect to the Poisson ($k = 10$).

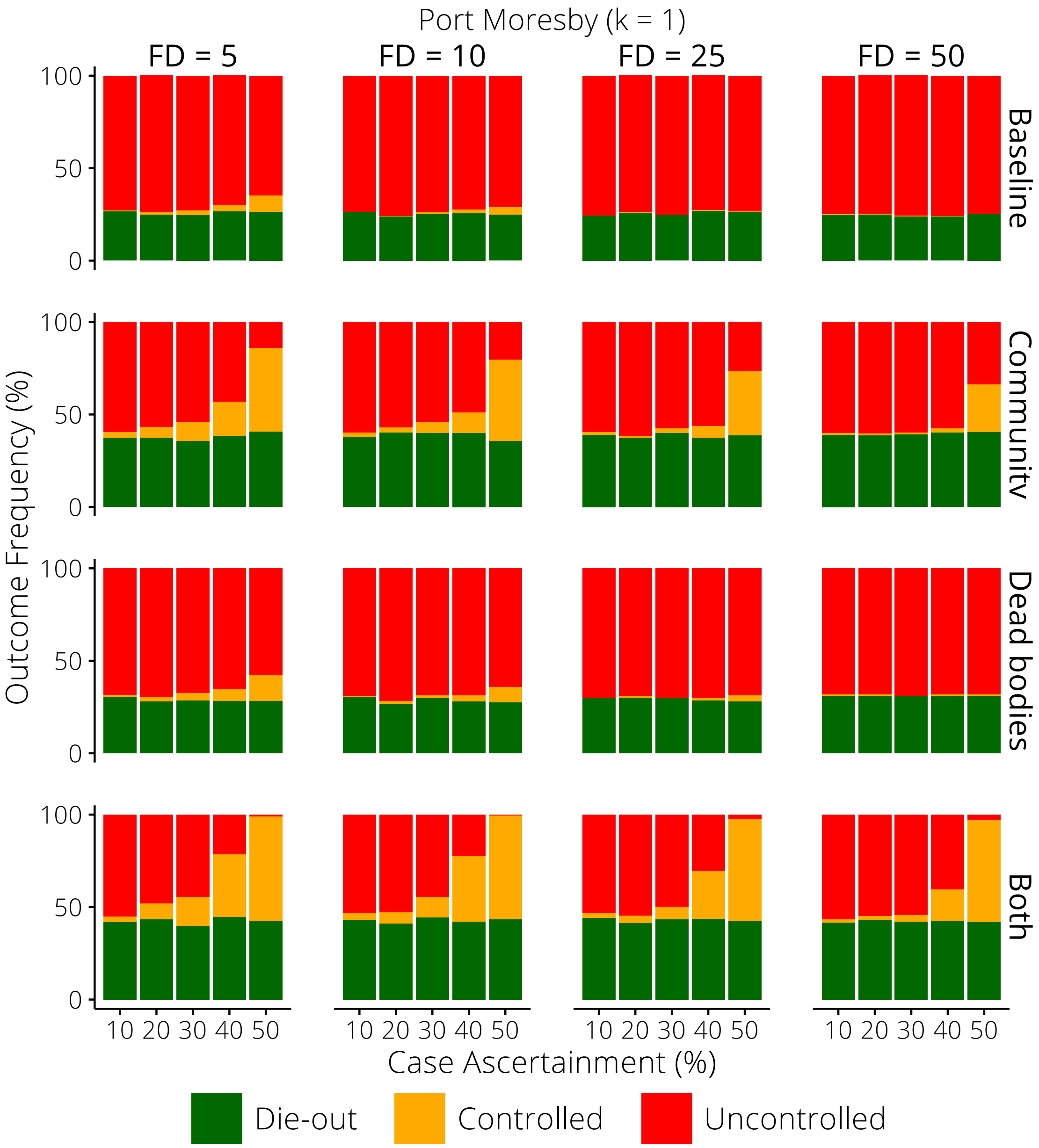

Figure 11: **Effect of behavioural interventions in Port Moresby region for $k = 1$.** The effect of reducing the force of infection in the community and/or from dead bodies by 25%, in the urban population of Port Moresby where community transmission is high, when the number of secondary cases are over-dispersed with respect to the Poisson ($k = 1$).

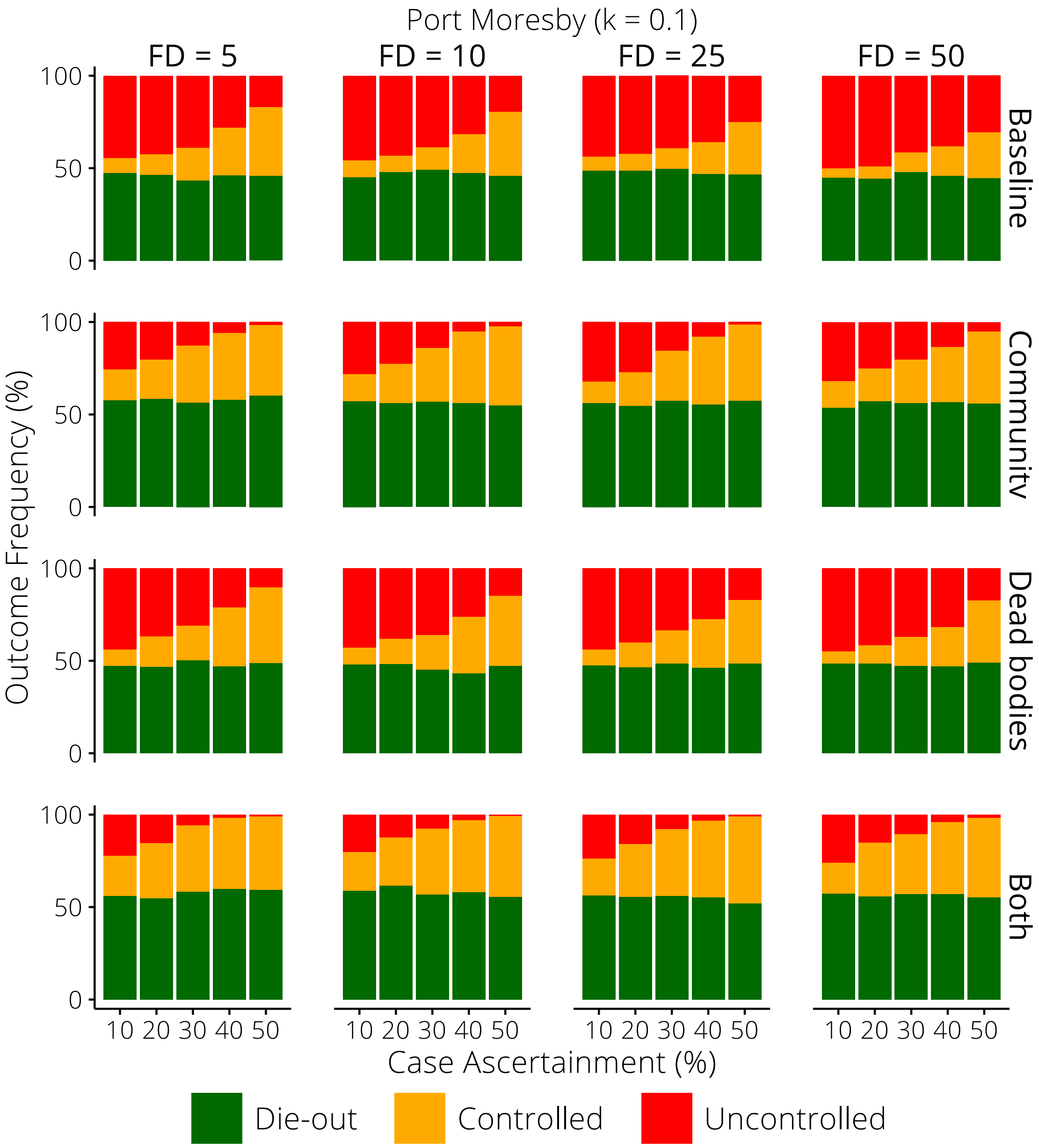

Figure 12: **Effect of behavioural interventions in Port Moresby region for $k = 0.1$.** The effect of reducing the force of infection in the community and/or from dead bodies by 25%, in the urban population of Port Moresby where community transmission is high, when the number of secondary cases are over-dispersed with respect to the Poisson ($k = 0.1$).
